# The current and potential health benefits of the National Health Service Health Check cardiovascular disease prevention programme in England: A microsimulation study

**DOI:** 10.1371/journal.pmed.1002517

**Published:** 2018-03-06

**Authors:** Oliver T. Mytton, Christopher Jackson, Arno Steinacher, Anna Goodman, Claudia Langenberg, Simon Griffin, Nick Wareham, James Woodcock

**Affiliations:** 1 MRC Epidemiology Unit, University of Cambridge, Cambridge, United Kingdom; 2 MRC Biostatistics Unit, University of Cambridge, Cambridge, United Kingdom; 3 London School of Hygiene and Tropical Medicine, London, United Kingdom; 4 Department of Public Health and Primary Care, University of Cambridge, Cambridge, United Kingdom; Edinburgh University, UNITED KINGDOM

## Abstract

**Background:**

The National Health Service (NHS) Health Check programme was introduced in 2009 in England to systematically assess all adults in midlife for cardiovascular disease risk factors. However, its current benefit and impact on health inequalities are unknown. It is also unclear whether feasible changes in how it is delivered could result in increased benefits. It is one of the first such programmes in the world. We sought to estimate the health benefits and effect on inequalities of the current NHS Health Check programme and the impact of making feasible changes to its implementation.

**Methods and findings:**

We developed a microsimulation model to estimate the health benefits (incident ischaemic heart disease, stroke, dementia, and lung cancer) of the NHS Health Check programme in England. We simulated a population of adults in England aged 40–45 years and followed until age 100 years, using data from the Health Survey of England (2009–2012) and the English Longitudinal Study of Aging (1998–2012), to simulate changes in risk factors for simulated individuals over time. We used recent programme data to describe uptake of NHS Health Checks and of 4 associated interventions (statin medication, antihypertensive medication, smoking cessation, and weight management). Estimates of treatment efficacy and adherence were based on trial data. We estimated the benefits of the current NHS Health Check programme compared to a healthcare system without systematic health checks. This counterfactual scenario models the detection and treatment of risk factors that occur within ‘routine’ primary care. We also explored the impact of making feasible changes to implementation of the programme concerning eligibility, uptake of NHS Health Checks, and uptake of treatments offered through the programme. We estimate that the NHS Health Check programme prevents 390 (95% credible interval 290 to 500) premature deaths before 80 years of age and results in an additional 1,370 (95% credible interval 1,100 to 1,690) people being free of disease (ischaemic heart disease, stroke, dementia, and lung cancer) at age 80 years per million people aged 40–45 years at baseline. Over the life of the cohort (i.e., followed from 40–45 years to 100 years), the changes result in an additional 10,000 (95% credible interval 8,200 to 13,000) quality-adjusted life years (QALYs) and an additional 9,000 (6,900 to 11,300) years of life. This equates to approximately 300 fewer premature deaths and 1,000 more people living free of these diseases each year in England. We estimate that the current programme is increasing QALYs by 3.8 days (95% credible interval 3.0–4.7) per head of population and increasing survival by 3.3 days (2.5–4.1) per head of population over the 60 years of follow-up. The current programme has a greater absolute impact on health for those living in the most deprived areas compared to those living in the least deprived areas (4.4 [2.7–6.5] days of additional quality-adjusted life per head of population versus 2.8 [1.7–4.0] days; 5.1 [3.4–7.1] additional days lived per head of population versus 3.3 [2.1–4.5] days). Making feasible changes to the delivery of the existing programme could result in a sizable increase in the benefit. For example, a strategy that combines extending eligibility to those with preexisting hypertension, extending the upper age of eligibility to 79 years, increasing uptake of health checks by 30%, and increasing treatment rates 2.5-fold amongst eligible patients (i.e., ‘maximum potential’ scenario) results in at least a 3-fold increase in benefits compared to the current programme (1,360 premature deaths versus 390; 5,100 people free of 1 of the 4 diseases versus 1,370; 37,000 additional QALYs versus 10,000; 33,000 additional years of life versus 9,000). Ensuring those who are assessed and eligible for statins receive statins is a particularly important strategy to increase benefits. Estimates of overall benefit are based on current incidence and management, and future declines in disease incidence or improvements in treatment could alter the actual benefits observed in the long run. We have focused on the cardiovascular element of the NHS Health Check programme. Some important noncardiovascular health outcomes (e.g., chronic obstructive pulmonary disease [COPD] prevention from smoking cessation and cancer prevention from weight loss) and other parts of the programme (e.g., brief interventions to reduce harmful alcohol consumption) have not been modelled.

**Conclusions:**

Our model indicates that the current NHS Health Check programme is contributing to improvements in health and reducing health inequalities. Feasible changes in the organisation of the programme could result in more than a 3-fold increase in health benefits.

## Abstract 2

Using a microsimulation model, Oliver Mytton and colleagues estimate the health benefits of the NHS Health Check programme in the UK and its potential impact on reducing health inequalities.

## Author summary

**Why was this study done?:**

- The National Health Service (NHS) Health Check programme is one of the first such programmes in the world, and there is uncertainty about the impact of the programme on health outcomes and inequalities.
- The programme is evolving, although there have been no estimates of the impact of possible changes in the way that the programme is delivered on health outcomes or inequalities.

**What did the researchers do and find?:**

- We developed a longitudinal microsimulation model to simulate the NHS Health Check programme and its impact on health, using epidemiological data for England and performance data for the programme.
- We estimated that the current NHS Health Check programme is preventing approximately 300 premature deaths (before 80 years) and resulting in an additional 1,000 people at age 80 years being free of cardiovascular diseases, dementia, and lung cancer each year in England. If risk of cardiovascular disease continues to decline, then these benefits will be attenuated by as much as a half.
- The benefits were greatest for people living in more deprived areas, and thus, the programme as a whole is reducing health inequalities.
- Making feasible changes to the delivery of the existing programme is likely to result in valuable improvements in health. For example, a strategy that combines extending eligibility to those with preexisting hypertension, extending the upper age of eligibility to 79 years, increasing uptake of health checks by 30%, and increasing treatment rates amongst eligible patients 2.5-fold (i.e., ‘maximum potential’ scenario) could result in at least a 3-fold increase in benefits compared to the current programme.

**What do these finding mean?:**

- The estimates of overall benefit are broadly in line with an estimate of likely benefit made prior to the programme’s introduction, which provides reassurance that the programme is meeting those expectations.
- The current practice of ensuring a higher attendance amongst people living in more deprived areas appears to be reducing health inequalities.
- There appears to be considerable scope to improve the health benefit of the programme by making feasible changes to its delivery, notably by ensuring those who are assessed and eligible for treatments receive appropriate treatment. Focusing on inviting previous nonattenders and widening the eligibility criteria to include those with an existing diagnosis of hypertension could also make a valuable contribution to increasing the health benefits of the programme.

## Introduction

The prevention of cardiovascular disease remains an important priority in the United Kingdom and elsewhere [1–4]. Cardiovascular disease accounts for around a quarter of all deaths and costs around £15 billion annually in the UK [5]. While there are a set of well-established actions to prevent cardiovascular disease, the uptake of these preventive interventions is suboptimal [6].

To address this, structured vascular risk assessment for adults aged 40–74 years without preexisting diabetes or cardiovascular disease (‘health checks’) was introduced in England in 2009 [7]. The programme sought to systematically identify individuals at risk of cardiovascular disease through a structured risk assessment and an offer of appropriate treatment, either pharmacological or behavioural. Now termed the National Health Service (NHS) Health Check programme, it consists of a defined set of interventions. While other components have been added over time, notably concerning alcohol, it retains a major focus on cardiovascular disease prevention [8]. The programme has been criticised for lacking evidence of benefit [9], and the overall health benefit it offers is unclear. While there have been trials of a ‘general health check’ in the past, many of these studies are old, with some pre-dating the introduction of more effective treatments like statins, and few, if any, of the interventions are comparable to the NHS Health Check programme [10–12].

Published evaluations of the current programme estimate benefit in terms of changes in cardiovascular risk factors (e.g., blood pressure), but these studies are prone to selection bias and do not estimate changes in ‘hard’ health outcomes [13,14]. Previous modelling studies have estimated the health benefit of a vascular check programme in England or the UK. One prior to the programme’s introduction sought to explicitly model the NHS Health Check programme and was based on an estimate of likely programme performance [7]. A second compared a universal (vascular) screening programme (based in part on the NHS Health Check programme) with concentrated screening and other population approaches to cardiovascular disease prevention [15]. The third study compared 7 different models for the delivery of a vascular ‘health check’ programme across 6 different European countries [16]. Whilst the vascular check programmes modelled in the latter 2 studies shared similarities with the NHS Health Check programme, neither explicitly modelled the NHS Health Check programme. None of these modelling studies make use of the more detailed emerging empirical data that characterise uptake by sociodemographic characteristics or the full range of data available on programme performance (e.g., referral to smoking cessation and weight management services) [13].

Despite concerns about overall benefit [9], the programme remains in place, is legally mandated as a universal programme [17], receives high-level political support [18,19], and is perceived favourably by patients [20,21]. Thus, the programme is likely to continue. A key focus is whether and how the existing programme could be more effective or (further) reduce health inequalities. Whilst there have been local evaluations of different approaches to programme delivery, we are not aware of any studies that have quantified the health impact and/or the effect on inequalities of making systemic changes to the programme’s delivery—for example, changing eligibility criteria, increasing attendance, or increasing uptake of treatments offered through the programme. Given that the programme is now established, it is an opportune time to review how the programme might evolve or change in order to improve impact.

We sought to address 2 questions. First, what is the health benefit and effect on health equity of the NHS Health Check programme as it is currently delivered in England? Second, we sought to understand the health benefits (or losses) that might accrue from making changes to the existing programme, considering eligibility criteria (widening or reducing eligibility); increasing the uptake of the programme (either generically or amongst high-risk groups); and improving uptake of treatments offered through the programme.

## Methods

**Fig 1:**
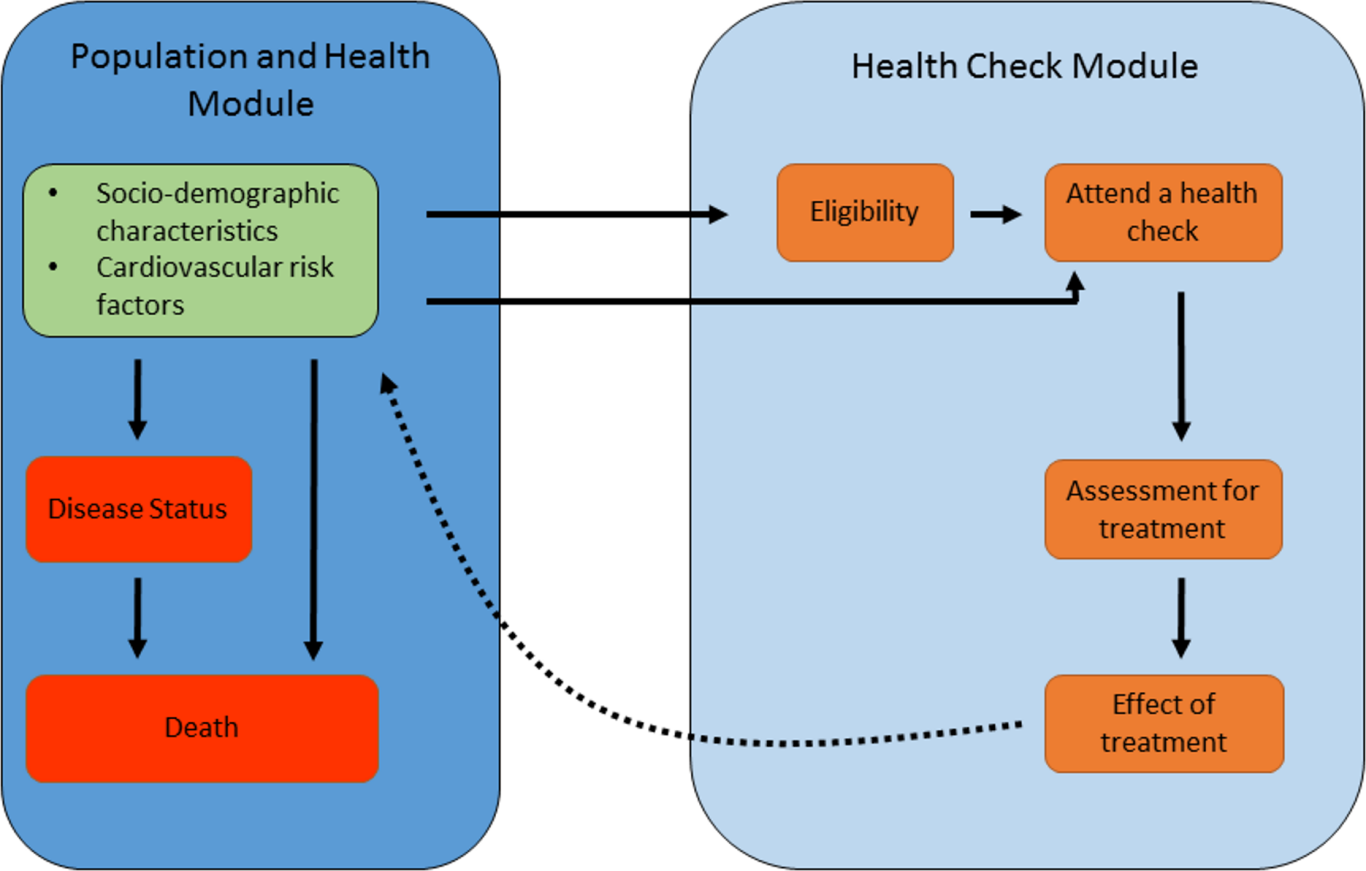
Outline of the microsimulation model.

We developed a microsimulation model to assess the effect of, and modifications to, the cardiovascular components of the NHS Health Check programme. The model consists of 2 modules (Fig 1). The first module (‘population and health’) describes the cardiovascular risk factors, disease status, and mortality of the population over time. The second module (‘Health Check’) simulates the different parts of the NHS Health Check: eligibility and attendance, assessment for treatment, and the effect of treatment. Further technical information on the methods are given as supplementary material (S1 Text), and the data inputs are summarised in Table 1.

**Table 1:** Summary of data inputs.

Part of model	Parameter	Data source/assumption
**Population**	Sociodemographic characteristics	Health Survey for England 2009–2012 [31].
	Health risk factors at baseline	Health Survey for England 2009–2012 [31].
	Change in risk factors over time	English Longitudinal Study of Ageing (ELSA) 1998–2012 [23].
**Disease Epidemiology**	Ischaemic heart disease (IHD) and stroke	Individual 10-year risk of cardiovascular disease was calculated using the QRisk2 score [45]. The 10-year risk was converted to annual risk based on routine data processed using DisMod. The likelihood of a new cardiovascular disease (CVD) event being a stroke or IHD was determined by age- and sex-specific estimates of incidence. Estimates of case fatality were derived from routine data processed using DisMod with further recalibration to account for raised mortality in the first year after diagnosis or presentation (i.e., myocardial infarction: 32% for men and 30% for women [34]; stroke: 7% for men and 5% for women aged under 80 years, 24% for men and 17% for women aged over 80 years) [20]. We also assumed for men and women that 58% and 44%, respectively, of IHD new presentations or diagnoses presented as a myocardial infarction [33] and that 60% of stroke presentations (QRisk2 includes both full strokes and transient ischaemic attacks) were a full stroke rather than a transient ischaemic attack. Routine data sources included the following: mortality statistics for England and Wales [27], the Health Survey for England (prevalence) [31], estimates on case fatality for IHD in England from linked mortality and hospital record data [46], and estimates on case fatality rates for stroke based on primary care records in the UK [47].
	Dementia	Individual 20-year risk of dementia was calculated using the cardiovascular risk factors, aging, and incidence of dementia risk (CAIDE) score [48]. The 20-year risk was converted to annual risk based on routine data processed using DisMod. Estimates of case fatality were derived from routine data processed using DisMod. Routine data sources included the following: the Cognitive Function and Aging Study II in England (incidence) [29] and a published audit of primary care records in the UK (relative risk of mortality) [30].
	Lung cancer	Annual estimates of incidence were based on routine data sources processed using DisMod. Lung cancer cases were attributed to smoking or not, based on published estimates of the proportion of lung cancer cases attributable to smoking in the UK [36]. Routine data sources included the following: mortality statistics for England and Wales [27] and cancer registry data for England (incidence) [26].
**National Health Service (NHS) Health Check programme**	Proportion of eligible population offered a health check	19.7% per year, based on published evaluation [49].
	Proportion of people offered a health check who attend	Estimates of uptake based on published evaluations and likelihood of attendance varied by age, sex, ethnicity, deprivation, smoking status, and QRisk2 score [13,14,50].
	Proportion of people getting a health check who are not eligible on the basis of a chronic condition	5% (95% credible interval [CrI] 2% to 8%): estimated by study team as no data were available.
**NHS Health Check—initiation of treatment**	Proportion of smokers at health check who are referred to smoking cessation therapy	3.6% (95% CrI 3.3% to 3.9%), assuming 6.8% of smokers (2,571/37,808) who had a health check were referred to smoking cessation, compared to 3.2% (9,944/310,034) of smokers who do not have a health check based on published programme evaluation [13].
	Proportion of obese people (BMI ≥ 30) at health check who are referred to weight management interventions	27.5% (95% CrI 26.9% to 28.1%), assuming 38.7% (12,430/32,133) of obese people who had a health check were referred to weight management, compared to 11.2% (4,441/39,774) of obese people who did not have a health check [13]. Only considers weight management, not the additional 31.1% of obese people who were referred to exercise, a group which is assumed to overlap substantially.
	Proportion who receive statins	QRisk2 < 20%: 2.05% (95% CrI 1.97 to 2.13) additional statin prescriptions in health check attenders versus nonattenders. QRisk2 ≥ 20%: 14.23% (95% CrI 13.71 to 14.76) additional statin prescriptions in health check attenders versus nonattenders. Based on published evaluation [13].
	Proportion of people with high blood pressure who receive antihypertensives	QRisk2 < 20%: 1.54% (95% CrI 1.46 to 1.62) additional antihypertensive prescriptions in health check attenders versus nonattenders. QRisk2 ≥ 20%: 2.48% (95% CrI 2.05 to 2.90) additional antihypertensive prescriptions in health check attenders versus nonattenders. Only individuals with hypertension at health check (defined as systolic blood pressure greater than 140 mmHg) were assumed to get antihypertensive treatment in either case. Based on published evaluation [13].
**Adherence to treatment**	Smoking cessation	We assumed that 100% of patients referred ‘adhere’ to treatment, as the treatment effectiveness estimates include those who are nonadherent.
	Weight management programme	We assumed 50% attend at least one session, i.e., assuming a lower real-world take-up rate than that in published trials of weight-loss interventions (e.g., 68% in Weight Loss Referrals for Adults in Primary Care [WRAP] trial) [51]. We assumed a 95% CrI of 30% to 70%.
	Statins	50% adherence to initial prescription (with a 95% CrI of 40% to 60%, from our assumption), based on published estimates [52–54]. An additional 5% (95% CrI 3% to 7%) of people taking statins are assumed to stop taking them each year (our assumption).
	Antihypertension medication	55% adherence (with a 95% CrI of 45% to 65%, from our assumption), based on published estimates [52,54,55]. An additional 5% (95% CrI 3% to 7%) of people on antihypertensives (AHTs) are assumed to stop taking them each year (our assumption).
**Treatment effectiveness**	Smoking cessation	Based on an evaluation of an English smoking cessation service, we assumed that 14.6% (95% CrI 13.1% to 16.1%) of those who are referred have quit at 1 year [56]. Relapse after quitting is modelled using ELSA data.
	Weight management effectiveness	Based on a published audit of weight management services in the UK, we assumed a mean BMI change of −1.5 kg/m^2^ by 1 year for everyone attending at least 1 session [57]. Lost weight assumed to be regained over 5 years, with BMI changes of −1.5, −0.9, −0.6, −0.3, and 0 at 1, 2, 3, 4, and 5 years after health check, respectively (our assumption).
	Statin effectiveness	Based on a meta-analysis of trials of efficacy of statins on cholesterol, we assumed a mean change of −1.22 (95% CrI −1.19 to −1.26) for men and −1.16 (95% CrI −1.10 to −1.23) for women in total cholesterol at 1 year [58] and a mean increase in high-density lipoprotein (HDL) at 1 year of 0.04 (95% CrI 0.028 to 0.052) for men and 0.036 (95% CrI 0.012 to 0.060) for women ([58]). Further adjustment was made to CVD risk for those on statins, reflecting published data on the efficacy of statin treatment (which was not adequately captured by changes in QRisk2 score) [58].
	Antihypertensive medication effectiveness	We assumed that those under 55 years used an angiotensin-converting-enzyme (ACE) inhibitor and those aged 55 years and over used calcium channel blockers. Based on a meta-analysis of efficacy of Ramipril on blood pressure, we assumed a mean change of −6.29/−4.14 mmHg (95% CrI −9.26 to −3.32/−5.81 to −2.48) [59]. Based on a meta-analysis of the efficacy of a calcium channel blocker on blood pressure, we assumed a mean change of −7.6/−3.1 mmHg (95% CrI −7.95 to −7.25/−2.75 to −3.45) for men and −9.0/−3.5 mmHg (95% CrI −8.68 to −9.32/−3.18 to −3.82) for women [60].

Published 95% confidence intervals have been used as estimates for 95% credible intervals.

### Population and health module

We simulated a closed cohort of 200,000 individuals aged 40–45 years representative of the English population, by sampling individuals from the Health Survey for England 2009–2012 to match the population structure (by gender and ethnicity) in the 2011 census [22]. Each individual had a set of demographic characteristics (age, sex, ethnicity, deprivation, and education) and a set of cardiovascular risk factors (blood pressure, smoking status, serum cholesterol, and body mass index). We modelled annual change in risk factors using the English Longitudinal Study of Aging (ELSA) (1998–2012) [23], which contains individual data on changes in cardiovascular risk factors over time and which (mostly) preceded the introduction of health checks. Changes in risk factors were estimated by matching each individual in our simulated population to one in the ELSA cohort with similar characteristics, with individuals being rematched as their risk factors changed. This nonparametric approach allowed us to generate trajectories that produced realistic results at the population level while also capturing between-individual heterogeneity.

#### Diseases

We estimated incidence and case fatality by age (year increments) and sex for 4 diseases: lung cancer, ischaemic heart disease, stroke, and dementia. We used QRisk2 to estimate risk of incident cardiovascular disease for each simulated individual based on sociodemographic and cardiovascular risk factors and the CAIDE score to estimate dementia incidence. These tools provide estimates of risk over a 10- and 20-year period, respectively, which were then converted to annual estimates of risk based on average estimates of population incidence estimated using DisMod II v1.05 [24] and routine data [25–31]. DisMod was used to generate annual estimates of incidence and case fatality from 2 or more estimates of routine data (e.g., prevalence and mortality).

QRisk2 estimates the risk of incident (first) diagnosis of cardiovascular disease (ischaemic heart disease, stroke, or TIA). New cardiovascular events were assigned at random to be either ischaemic heart disease or a stroke, reflecting the relative proportions of these events by age. In doing this, we assumed that the ratio of strokes to TIAs was 60:40 [32].

We assumed that 58% of new diagnoses of ischaemic heart disease for men and 44% for women were an acute myocardial infarction [33]. We further assumed that a proportion of acute events (32% for men and 33% for women for acute myocardial infarction; 7% for men and 5% for women under the age of 80 years, and 24% and 17% for men and women aged 80 years and over) were fatal [34,35]. Making allowance for acute fatality, we recalibrated the annual estimates of case fatality, from DisMod based on routine data sources, to account for acute mortality, i.e., we modelled a higher fatality in the year of diagnosis or presentation. Unadjusted estimates of case fatality by age and sex for dementia and lung cancer from DisMod were used.

For smoking, we calculated separate incidence rates for smokers and nonsmokers using DisMod based on reported population attributable fractions [36].

Whilst dementia was not a focus in the original programme [7], it has subsequently been included. Dementia shares many of the same risk factors as ischaemic heart disease and stroke, and vascular pathology in the brain is associated with dementia [37–39]. It is thought that dementia risk is modifiable and that recent reductions in incidence might be attributable to better management of cardiovascular risk [29,40,41]. On this basis, dementia was included in the model, although to date there is no evidence from randomised clinical trials showing that dementia risk can be reduced.

Once an individual developed a disease, it was assumed that he or she had the disease for life, adopting the appropriate mortality risk. Mortality from all other causes was estimated using standard life tables [42], after adjusting for cause-specific mortality within the model. The model proceeds in annual increments, with individuals followed until death or 100 years of age.

At the beginning of the simulation, a proportion of the population (based on estimates from DisMod) were assumed to have ischaemic heart disease or stroke. The probability of having cardiovascular disease at baseline depended on the QRisk2 score at baseline. We assumed that nobody had dementia or lung cancer at baseline.

### Health check module

Simulated individuals were eligible for a health check based on their age and their disease status (absence of diabetes, cardiovascular disease, and hypertension), reflecting current eligibility criteria. We assumed that the annual probability of an eligible individual being offered a health check was 0.197, based on national programme data [43]. Thus, on average, a person would be offered a health check once every 5 years.

We assumed that the likelihood of attendance was determined by sociodemographic characteristics (age, ethnicity, and area-level deprivation) and cardiovascular risk factors (smoking status and QRisk2), based on programme data[13], and (in the absence of long-term data) we assumed that past attendance did not affect future attendance. We also assumed that 5% of ineligible individuals attended for an NHS Health Check, e.g., via a drop-in clinic in a pharmacy.

We assumed that individuals could be offered 1 or more of 4 treatments: statins, antihypertensives, smoking cessation, and weight management [44]. Assumptions regarding who got treated, adherence to treatment, and the effect of treatment are summarised in Table 1. We assumed no interaction between treatments.

For all individuals receiving statins, antihypertensives, or weight management, a counterfactual trajectory for each risk factor, without treatment after an NHS Health Check, was simulated using the ELSA data. A treatment trajectory was then estimated by adjusting the counterfactual trajectory to represent the effect of treatment. For example, if a woman was compliant with statin therapy (initiated because of a health check), her cholesterol level would be 1.16 mmol/L lower than her background (or ‘counterfactual’) cholesterol. For individuals who quit smoking after attending a smoking cessation programme, their risk of relapse and future smoking status were estimated by matching to individuals in the ELSA dataset and adjusting for published evidence on quitting and relapsing [61]. In all cases, we have effectively modelled a decline in treatment effectiveness over time due either to reduced adherence or relapse (see Table 1) such that the health benefits over time may appear to be less than expected from trial data but should be more akin to the benefit attributable to the NHS Health Check programme in the real world.

The simulation model was written in Python (version 2.7.6). The full source code is available under licence from a GitHub repository (https://github.com/chjackson/healthchecks).

### Modelled health benefits

In all cases, the effect of changes in risk factors on health was modelled through the respective disease risk scores for cardiovascular disease and dementia. For smoking, we estimated separate incidence rates for smokers and nonsmokers, as described previously. Body mass index was assumed to have a direct effect on QRisk2 score (rather than through changes in blood pressure and cholesterol).

### Model calibration and validation

We sought to identify the most suitable data for our model and focused on developing the aspects of the model that are most important for the scenarios we sought to explore. During the development of the model, we undertook a series of checks to ensure the model was accurately simulating the health check process (i.e., attendance and treatment uptake) and outputs (e.g., blood pressure and QRisk2) by comparing model outputs with empirical data. We also compared trial data for blood pressure medication and statins with published trial data on treatment efficacy. Changes in QRisk2 due to changes in serum cholesterol did not accurately capture the reduction in risk for statin treatment reported in trials, as would be expected, as statins reduce cardiovascular risk by other means (e.g., reducing inflammation) [62]. Consequently, we made a further adjustment to cardiovascular disease (CVD) risk for people who were started on a statin by calibrating the modelled reduction in CVD event rates over 5 years achieved by statin so that it was equal to the value observed in trials [58]. To validate the population and health module, we compared estimates of mortality for ischaemic heart disease and stroke by sex—produced by this part of the model—with published estimates of mortality based on death certification [25]. There was reasonably close agreement (S1 Data).

### Scenarios

First, we compared the present NHS Health Check programme (assuming it continues to operate in its present format) to a counterfactual in which no NHS Health Check programme operated. Second, we explored different scenarios for how the NHS Health Check programme could evolve in the future, considering 3 areas: eligibility criteria, uptake of the programme, and treatment.

We considered 4 scenarios in which the eligibility criteria would change: (1) extending the programme to invite those who already have a diagnosis of hypertension, (2) increasing the age at which individuals are first invited for a health check from 40 years to 50 years of age, (3) increasing the upper age at which individuals may be invited to attend the programme from 74 years to 79 years, and (4) changing both the upper and lower age criteria, such that persons aged 50–79 years would be invited.

We considered 5 scenarios in which the likelihood of attendance was increased by 30%: (1) for everyone invited, (2) for people living in the most deprived areas (bottom quintile group), (3) for smokers, (4) for those at high cardiovascular risk (QRisk2 > 20%), and (5) for nonattenders (people who did not attend in the past 5-year cycle).

We considered 5 scenarios in which the likelihood of receiving treatment amongst those eligible at assessment was increased 2.5-fold for statins alone, antihypertensives alone, smoking cessation alone, weight management alone, and all treatments. The value of 2.5 was selected in light of evidence from Tower Hamlets, London, indicating that the proportion of high-risk (QRisk2 > 20%) health check attenders additionally prescribed statins was around 36%, approximately 2.5 times higher than the national figure of 14% [63]. Whilst it is unclear what the maximum feasible uptake of different treatments is, we have modelled the same relative increase for all treatments and note that programme data suggest that such increases appear, on paper, to be feasible.

Finally, to demonstrate the combined benefit of increasing uptake and improving delivery of health checks, we simulated the effect of simultaneously widening eligibility to include those with a diagnosis of hypertension, increasing attendance by 30% for everyone and increasing all treatments by 2.5-fold, which we will term a ‘maximum potential’ scenario.

### Outcomes

Primary outcomes are total incident cases prevented (by age 80 years), premature deaths prevented (<80 years), change in quality-adjusted life years (QALYs), and change in survival. We also report incident events prevented by age 100 years (as well as providing a breakdown by disease category) and deaths prevented before 75 years of age. We provide estimates of the total additional quality-adjusted years lived and years lived for the studied cohort of 1,000,000 people and estimates per head of population. We use the latter metric as the primary means to describe the effect of health inequalities, as these measures make allowance for different population sizes.

Standard EQ-5D disutility weights for age, deprivation, and disease status were used to estimate QALYs [64].

To provide a comparison with other published estimates that describe the number of events avoided in England each year, we multiply our estimates for events avoided over the life of the cohort by 0.73 (there are approximately 730,000 adults aged 40 in any given year in England). This assumes the benefits observed for the current population in any given year are comparable to the benefits that the cohort (aged 40–45 years) experience longitudinally.

We also provide estimates for those living in the most and least deprived areas (expressed per head of population to standardise for differences in population size). Each simulated individual adopted the quintile group of the sampled individual from the Health Survey for England (based on the Index of Multiple Deprivation for the area of residence).

### Uncertainty analyses

We used probabilistic methods to account for uncertainty in parameters entered into the model. We assigned a probability distribution, rather than a fixed value, for each input parameter. We then ran our model 100 times, each time sampling each parameter from its stated distribution. This yielded 100 output values, from which we calculated a mean and 95% credible intervals. The number of samples was chosen such that the sampling error in mean outcomes over individuals (due to simulating a finite number of individuals) was small (<5% of that due to parametric uncertainty). In each run, we simulated 200,000 individuals, effectively sampling 20 million individuals for each modelled scenario.

We also undertook ‘value of information analyses’ to identify which sources of parametric uncertainty were contributing the most to uncertainty in the results. These are calculated by estimating the standard deviation for the result if the exact value of the parameter of interest were to be learnt. This can be compared to the width of the original 95% credible interval to describe the potential value of obtaining perfect information on that parameter. All parameters with uncertainty or credible intervals were considered. We repeated the ‘value of information analysis’ for 2 of the scenarios: our first scenario (comparing the NHS Health Check programme to a counterfactual in which no programme operated) and the ‘maximum potential’ scenario.

### Sensitivity analyses

We also undertook the following sensitivity analyses:

1. Future attendance was independent of past attendance
  The model assumes that attendance at a health check is independent of past attendance record. Given the programme has been in existence for less than 10 years, there is limited long-term data to assess the extent to which past attendance predicts future attendance. However, we note that for similar programmes (i.e., screening programmes) past attendance can be a predictor of future attendance [65–67]. We thus modelled the current programme under the assumption that likelihood of attendance was greater (average of 70% per 5-year cycle) if somebody attended after his or her most recent offer of a health check and less if he or she did not (average 30%). We further tested the effect of this assumption on the scenario concerned with increasing invitations to nonattenders.
2. Future changes in CVD incidence and fatality
  The model assumes that present incidence of and case fatality from CVD continue. However, age-standardised mortality for ischaemic heart disease and stroke has fallen over the past 50 years [68], and while there is uncertainty about the nature of future trends, particularly in light of the rising prevalence of obesity and diabetes[69], it seems likely that these trends will continue at least in the short to medium term. We assumed that the recently observed trends would continue for the next 20 years before plateauing. We assumed that the incidence of ischaemic heart disease (IHD) would fall by 4.8% for men and 4.5% for women per year [34] and that stroke incidence would fall by 4.0% per year [47]. We assumed that the case fatality for IHD would fall by 3.6% per year [34] and that the case fatality for stroke would fall by 6.0% per year [35]. We did not model declines for a longer period of time as the observed trends were for a short time period and there is considerable uncertainty about long-term trends.
3. Uncertainty in population cardiovascular risk (at baseline)
  To understand the extent to which our estimates would change if the model’s estimate of cardiovascular risk in the cohort was too high or too low (i.e., reflect uncertainty in our estimate of average risk in the population), we reran our model by multiplying the QRisk2 score by a value chosen from a log-normal distribution with 95% quantiles of 0.8 and 1.2. The value was applied to each simulated individual in any given run of the model (with different values drawn from the distribution being applied to different runs).

## Results

The baseline characteristics of the population aged 40–45 years are shown in Table 2. In the absence of the NHS Health Check programme, we estimate that per million people aged 40–45 years at baseline in the 60 years of follow-up, there will be 355,000 diagnoses of IHD, 184,000 diagnoses of stroke, 147,000 diagnoses of dementia, 148,000 diagnoses of lung cancer, and 405,000 premature (<80 years) deaths.

**Table 2:** Baseline characteristics of the whole population aged 40–45 years and those who go on to participate in the health check programme.

	Everyone aged 40–45 years	People eligible for a health check at least once (85%)	People who go on to attend at least 1 health check (77%)
**Gender**			
Male	50.4%	51.1%	51.6%
Female	49.6%	48.9%	48.4%
**Ethnicity**			
White	86.1%	86.2%	86.2%
Indian	2.5%	2.6%	2.7%
Pakistani	2.4%	2.4%	2.4%
Caribbean	1.6%	1.5%	1.5%
African	2.2%	2.2%	2.1%
Other	5.1%	5.1%	5.1%
**Deprivation**			
1 (least deprived)	22.1%	23.5%	23.0%
2	22.9%	22.3%	22.2%
3	21.3%	22.6%	22.2%
4	19.8%	19.3%	19.4%
5 (most deprived)	13.9%	12.3%	13.1%
**Education**			
≥10 years or equivalent	44.9%	45.9%	45.7%
7–9 years	45.7%	45.1%	45.2%
≤6 years	9.4%	9.0%	9.1%
**QRisk2**	2.9 (1.1 to 3.8)	2.5 (1.0 to 3.3)	2.6 (1.1 to 3.4)
**QRisk2 >20**	0.4%	0.2%	0.2%
**QRisk2 10–20**	2.2%	0.5%	0.9%
**Systolic blood pressure**	122.6 (112.5 to 131.5)	120.7 (111.5 to 128)	120.9 (112.5 to 128.5)
**Diastolic blood pressure**	75.2 (68.0 to 81.5)	73.6 (67.0 to 80.0)	73.9 (68.0 to 80.0)
**Blood pressure >140/90**	14.2%	10.3%	10.8%
**Treated hypertension**	4.6%	0.0%	1.1%
**Cholesterol**	5.5 (4.8 to 6.1)	5.5 (4.8 to 6.1)	5.5 (4.8 to 6.1)
**TC/HDL**	4.0 (3.0 to 4.9)	4.0 (2.9 to 4.8)	4.0 (3.0 to 4.8)
**BMI (kg/m**^**2**^**)**	27.5 (24.2 to 30.2)	27.1 (23.9 to 29.6)	27.1 (24.2 to 29.8)
**Obese**	27.1%	23.0%	23.6%
**HbA1c**	5.6 (5.3 to 5.7)	5.5 (5.3 to 5.7)	5.5 (5.3 to 5.7)
**HbA1c >6.5%**	3.4%	4.0%	4.4%
**Diabetes**	3.3%	0.0%	1.0%
**Smoking**			
Never	52.6%	52.8%	53.3%
Ex	22.8%	22%	22.4%
Current	24.6%	25.2%	24.3%

Abbreviations: HbA1c, haemoglobin A1c; HDL, high-density lipoprotein; TC, total cholesterol. Mean and interquartile range are given for continuous variables. Deprivation quintile groups are based on the Index of Multiple Deprivation for the area of residence. QRisk2 is the 10-year risk of cardiovascular disease [45].

### Current programme

At some point during their 35-year period of potential eligibility, we estimate that 85% of the cohort will be eligible for and 80% will attend for at least 1 health check (Table 3). Whilst the majority (81% of the population) will be eligible for treatment at some point, only a minority (27% of the population) are offered treatments through the NHS Health Check programme. The most common treatment offered is weight management.

**Table 3:** Summary of process measures and outcomes for the present National Health Service (NHS) health check programme.

**Eligibility and uptake (for whole population)**	
Eligible for an NHS Health Check at any time (%)	85.1
Have one or more NHS Health Checks (%)	79.7
Mean health checks (per person)	1.9
**Treatment (as a proportion of the whole population)**	
Eligible for an NHS Health Check and any treatment (%)	81.0
Attended for an NHS Health Check and eligible for any treatment (%)	73.3
Offered any treatment (%)	26.6
Offered statins through an NHS Health Check (%)	8.5
Offered antihypertensives through an NHS Health Check (%)	3.3
Referrals for a weight loss programme through an NHS Health Check (%)	17.6
Referrals for smoking cessation services through an NHS Health Check (%)	1.2
Treated with statins through an NHS Health Check (%)	4.3
Treated with antihypertensives through an NHS Health Check (%)	1.8
Attended a weight loss programme after referral from an NHS Health Check (%)	10.0
Attended smoking cessation after referral from an NHS Health Check (%)	0.1
**Cases prevented by age 80 (per million)**	
IHD	1,089 (817 to 1,367)
Stroke	525 (414 to 671)
Dementia	135 (72 to 190)
Lung cancer	90 (36 to 147)
Additional people living free of one of the diseases listed above at age 80 years	1,371 (1,101 to 1,685)
**Cases prevented by age 100 (per million)**	
IHD	1,296 (898 to 1,730)
Stroke	679 (494 to 958)
Dementia	125 (32 to 222)
Lung cancer	175 (103 to 259)
Additional people living free of one of our diseases at age 100 years	1,235 (956 to 1,566)
**Premature deaths prevented (per million)**	
<75 years	246 (182 to 331)
<80 years	386 (291 to 499)
**Quality-adjusted life gained over 60 years of follow-up**	
Total (QALYs for whole population)	10,300 (8,170 to 12,900)
Days per head of population	3.8 (3.0 to 4.7)
Days per eligible person	4.3 (3.4 to 5.4)
Days per person screened at least once	4.7 (3.8 to 6)
Days per health check	2.0 (1.6 to 2.5)
Days per head (most deprived quintile group)	5.1 (3.4 to 7.1)
Days per head (least deprived quintile group)	3.3 (2.1 to 4.5)
**Life gained over 60 years of follow-up**	
Total (years for whole population)	9,700 (6,880 to 11,300)
Days per head of population	3.3 (2.5 to 4.1)
Days per eligible person	3.7 (2.8 to 4.8)
Days per person screened at least once	4.1 (3.2 to 5.2)
Days per health check	1.7 (1.4 to 2.2)
Days per head (most deprived quintile group)	4.4 (2.7 to 6.5)
Days per head (least deprived quintile group)	2.8 (1.7 to 4.0)

Abbreviations: HC, an NHS Health Check; IHD, ischaemic heart disease. Deprivation quintile groups are based on the Index of Multiple Deprivation score for the area of residence. Cases prevented are all cases prevented when following a cohort of 1 million adults aged 40–45 years until either 80 or 100 years of age. Premature deaths prevented are all-cause deaths prevented before age 75 or 80 years, when following a cohort of 1 million adults aged 40–45 years until 80 years of age. Quality-adjusted life gained and life gained are over the 60 years of follow-up, i.e., the remaining lifetime of the cohort. Estimates of 95% credible intervals (due to parameter uncertainty) are shown in parentheses. The 95% credible intervals are the 2.5th and 97.5th percentile of all estimates from 100 simulations.

We estimate that the NHS Health Check programme prevents 390 (95% credible interval [CrI] 290 to 500) premature deaths before 80 years of age and results in an additional 1,370 (95% CrI 1,100 to 1,690) people being free of disease (IHD, stroke, dementia, and lung cancer) at age 80 years per million people aged 40–45 years at baseline.

Over the life of the cohort (i.e., followed from 40–45 years to 100 years), we estimate the changes result in an additional 10,000 (95% CrI 8,200 to 13,000) QALYs and an additional 9,000 (6,900 to 11,300) years of life. This is equivalent to 3.8 (3.0 to 4.7) days of quality-adjusted life per head of population and an increase in survival of 3.3 (2.5 to 4.1) days per head of population. The increase in quality-adjusted life (3.8 days) is greater than the increase in survival (3.3 days), i.e., the intervention results in compression of morbidity.

Assuming there are 730,000 people aged 40 years in England each year, this would equate to approximately 300 fewer premature deaths before 80 years of age, 1,000 more people living free of CVD, dementia, and lung cancer at age 80 years, 7,500 additional QALYs, and 6,600 extra years of life each year in England.

### Changing eligibility criteria

The effect of making changes to the eligibility criteria is shown in Table 4. All the estimates for health impacts (cases prevented, deaths prevented, QALYs, and survival) are changes relative to the current NHS Health Check programme. To estimate changes in these outcomes relative to a counterfactual with no NHS Health Check programme, these values should be added to the corresponding values in Table 3.

**Table 4:** Effect of new eligibility criteria on process and outcome measures of the National Health Service (NHS) health check programme.

	New eligibility criteria			
	Include people with hypertension	Starting age 50 years	Upper age of eligibility 79 years	Starting age (50 years) and upper eligibility age (79 years)
**Eligibility and uptake**				
Eligible for HC at any time (%)	92.5	79.7	85.1	79.8
Have one or more HCs (%)	85.3	76.8	79.8	77.2
Mean HCs per head of population	2.1	1.7	2.1	1.9
**Treatment (proportion of whole population)**				
Eligible for HC and any treatment (%)	88.3	76.9	81.0	76.9
Attended HC and were eligible for any treatment when attending (%)	79.0	71.5	74.5	72.9
Offered any treatment through HC (%)	29.6	25.1	29.5	28.2
Offered statins through HC (%)	9.4	8.0	10.7	10.3
Offered antihypertensives through HC (%)	3.7	3.0	3.8	3.5
Referred to a weight loss programme through HC (%)	19.9	16.6	18.9	18
Referred to smoking cessation services through HC (%)	1.3	1.1	1.3	1.1
Treated with statins through HC (%)	4.8	4.1	5.4	5.3
Treated with antihypertensives through HC (%)	2.0	1.6	2.1	1.9
Attended a weight loss programme after HC referral (%)	11.3	9.4	10.9	10.3
Attended smoking cessation services after HC referral (%)	0.1	0.1	0.1	0.1
**Additional cases prevented by age 80 (per million)**				
IHD	162 (91 to 232)	−38 (−81 to −4)	136 (93 to 184)	97 (40 to 160)
Stroke	76 (36 to 118)	−19 (−49 to 5)	68 (30 to 111)	49 (−1 to 99)
Dementia	16 (−11 to 45)	−13 (−36 to 8)	0 (−5 to 0)	−13 (−36 to 8)
Lung cancer	12 (−5 to 30)	−8 (−25 to 6)	5 (−1 to 16)	−3 (−21 to 16)
Additional people living free of one of our diseases at age 80 years	194 (114 to 264)	−53 (−94 to −7)	185 (111 to 248)	133 (48 to 203)
**Additional cases prevented by age 100 (per million)**				
IHD	179 (110 to 279)	−36 (−76 to 0)	339 (226 to 442)	303 (198 to 411)
Stroke	88 (41 to 155)	−17 (−52 to 8)	188 (108 to 272)	171 (90 to 253)
Dementia	13 (−21 to 42)	−16 (−38 to 14)	−25 (−45 to −4)	−41 (−73 to 0)
Lung cancer	23 (3 to 51)	−13 (−39 to 6)	16 (−3 to 39)	3 (−28 to 35)
Additional people living free of one of our diseases at age 100 years	160 (89 to 219)	−44 (−81 to −4)	330 (235 to 436)	286 (183 to 392)
**Additional premature deaths prevented (per million)**				
<75 years	34 (9 to 65)	−15 (−39 to 1)	0 (0 to 0)	−15 (−39 to 1)
<80 years	57 (19 to 99)	−18 (−44 to 5)	33 (9 to 68)	15 (−24 to 49)
**Additional quality-adjusted life gained over the 60 years of follow-up**				
Total (QALYs for whole population)	1,400 (688 to 2,010)	−604 (−1,090 to −127)	1,480 (868 to 2,050)	876 (185 to 1,670)
days per head of population	0.5 (0.3 to 0.7)	−0.2 (−0.4 to 0.0)	0.5 (0.3 to 0.7)	0.3 (0.1 to 0.6)
days per eligible person	0.6 (0.3 to 0.8)	−0.2 (−0.4 to 0.0)	0.6 (0.4 to 0.9)	0.4 (0.1 to 0.8)
days per person screened at least once	0.6 (0.3 to 0.9)	−0.2 (−0.4 to 0.0)	0.7 (0.4 to 0.9)	0.5 (0.2 to 0.9)
days per health check	0.0 (−0.1 to 0.2)	0.1 (0.0 to 0.2)	0.1 (0.0 to 0.2)	0.2 (0.0 to 0.3)
days per head (most deprived quintile group)	0.9 (0.2 to 1.7)	−0.3 (−0.8 to 0.4)	0.5 (0.1 to 1)	0.2 (−0.5 to 1)
days per head (least deprived quintile group)	0.4 (0 to 0.8)	−0.2 (−0.5 to 0.1)	0.6 (0.2 to 1)	0.4 (−0.2 to 0.9)
**Additional life gained over the 60 years of follow-up**				
Total (years for whole population)	1,220 (577 to 1,860)	−511(−29.5 to −1,060)	1,320 (706 to 1,870)	813 (83.5 to 1,640)
days per head of population	0.4 (0.2 to 0.7)	−0.2 (−0.4 to 0.0)	0.5 (0.3 to 0.7)	0.3 (0.0 to 0.6)
days per eligible person	0.5 (0.2 to 0.7)	−0.2 (−0.4 to 0.0)	0.6 (0.3 to 0.8)	0.4 (0.1 to 0.8)
days per person screened at least once	0.5 (0.2 to 0.8)	−0.1 (−0.3 to 0.0)	0.6 (0.3 to 0.9)	0.5 (0.2 to 0.8)
days per health check	0.0 (−0.1 to 0.2)	0.1 (0.0 to 0.2)	0.1 (0.0 to 0.2)	0.1 (0.0 to 0.3)
days per head (most deprived quintile group)	0.8 (0.1 to 1.7)	−0.2 (−0.8 to 0.4)	0.5 (0.0 to 1.0)	0.2 (−0.5 to 1.1)
days per head (least deprived quintile group)	0.3 (0.0 to 0.8)	−0.2 (−0.5 to 0.1)	0.5 (0.2 to 1.0)	0.4 (−0.2 to 0.8)

Abbreviations: HC, an NHS Health Check; IHD, ischaemic heart disease; QALY, quality-adjusted life year. Deprivation quintile groups are based on the Index of Multiple Deprivation score for the area of residence. Health outcomes (cases prevented, premature deaths prevented, days of quality-adjusted life, and days of life gained) are expressed relative to the existing programme. Cases prevented are all cases prevented when following a cohort of 1 million adults aged 40–45 years until either 80 or 100 years of age. Premature deaths prevented are all-cause deaths prevented before age 75 or 80 years, when following a cohort of 1 million adults aged 40–45 years until 80 years of age. Additional quality-adjusted life gained and additional life gained are over the 60 years of follow-up, i.e., the remaining lifetime of the cohort. Estimates of 95% credible intervals (due to parameter uncertainty) are shown in parentheses. The 95% credible intervals are the 2.5th and 97.5th percentile of all estimates from 100 simulations.

Options associated with improvements in population health were opening the programme to people with a diagnosis of hypertension and extending the upper age of cutoff to 79 years. Increasing the starting age for eligibility from 40 years to 50 years was associated with a reduction in population health. A hybrid approach, raising the starting age and raising the upper age cutoff, was also associated with an improvement in population health (the loss from increasing the starting age being offset by the gain from increasing the upper age cutoff).

### Increasing attendance

Increasing attendance is associated with improvements in indices of population health (Table 5). Increasing attendance for everyone (by 30%) results in the greatest improvements in population health, although selective approaches (e.g., increasing attendance amongst those at high risk of CVD by 30% or increasing the likelihood of invitation to those who did not attend in the past 5-year cycle) may yield relatively large gains in population health for fewer additional health check appointments.

**Table 5:** Effect of increasing attendance on process and outcome measures of the National Health Service (NHS) health check programme (*n* = 1,000,000).

	Uptake increased by 30% for everyone	Uptake increased by 30% for the most deprived quintile group	Uptake increased by 30% for smokers	Uptake increased by 30% for those at high risk of CVD^±^	Increase likelihood of offer of a health check to previous nonattenders by 30%
**Eligibility and uptake**					
Eligible for HC at any time (%)	85.1	85.1	85.1	85.1	85.1
Have one or more HCs (%)	83.0	80.0	80.4	80.4	81.4
Mean HCs per head of population	2.2	1.9	2.0	2.0	2.1
**Treatment (proportion of whole population)**					
Eligible for HC and any treatment (%)	81.0	81.0	81.0	81.0	81.0
Attended HC and were eligible for any treatment when attending (%)	77.0	73.6	74.1	74.3	75.3
Offered any treatment through HC (%)	29.8	27	27.3	27.7	28.4
Offered statins through HC (%)	9.6	8.6	8.7	9.2	9.0
Offered antihypertensives through HC (%)	3.8	3.4	3.4	3.5	3.6
Referred to a weight loss programme through HC (%)	19.8	17.9	18	18.1	18.9
Referred to smoking cessation services through HC (%)	1.4	1.2	1.4	1.3	1.3
Treated with statins through HC (%)	4.9	4.4	4.4	4.7	4.6
Treated with antihypertensives through HC (%)	2.1	1.8	1.9	1.9	2.0
Attended a weight loss programme after HC referral (%)	11.4	10.2	10.2	10.3	10.8
Attended smoking cessation services after HC referral (%)	0.2	0.1	0.2	0.1	0.1
**Additional cases prevented by age 80**					
IHD	149 (60 to 244)	23 (−10 to 65)	42 (2 to 97)	93 (37 to 154)	90 (28 to 154)
Stroke	78 (25 to 146)	12 (−11 to 45)	25 (−10 to 64)	49 (12 to 91)	43 (−5 to 96)
Dementia	29 (−3 to 63)	6 (−9 to 21)	7 (−6 to 28)	2 (−10 to 14)	19 (−4 to 43)
Lung cancer	15 (−12 to 44)	2 (−5 to 13)	16 (−9 to 45)	3 (−9 to 15)	9 (−15 to 30)
Additional people living free of one of our diseases at age 80 years	200 (82 to 282)	28 (−13 to 66)	58 (19 to 107)	104 (42 to 164)	122 (49 to 191)
**Additional cases prevented (by age 100)**					
IHD	172 (74 to 290)	26 (−16 to 64)	41 (−16 to 94)	125 (50 to 207)	95 (11 to 170)
Stroke	99 (19 to 171)	15 (−18 to 57)	26 (−14 to 75)	72 (15 to 140)	53 (−12 to 118)
Dementia	34 (−22 to 83)	5 (−19 to 28)	5 (−22 to 41)	−7 (−36 to 15)	25 (−21 to 73)
Lung cancer	30 (−5 to 76)	3 (−12 to 22)	31 (−3 to 74)	9 (−11 to 35)	19 (−14 to 53)
Additional people living free of one of our diseases at age 100 years	181 (63 to 308)	26 (−7 to 63)	41 (−8 to 88)	100 (39 to 180)	107 (24 to 191)
**Additional deaths prevented (all cause)**					
<75 years	40 (6 to 76)	6 (−12 to 21)	18 (−10 to 45)	18 (−10 to 46)	25 (−1 to 56)
<80 years	60 (14 to 115)	10 (−9 to 33)	22 (−11 to 55)	30 (0 to 64)	37 (2 to 79)
**Additional quality-adjusted life gained over the 60 years of follow-up**					
Total (QALYs for whole population)	1720 (781 to 2,760)	268 (−128 to 658)	594 (−27.1 to 1,140)	805 (284 to 1,280)	990 (302 to 1,740)
days per head of population	0.6 (0.3 to 1)	0.1 (0 to 0.2)	0.2 (0 to 0.4)	0.3 (0.1 to 0.5)	0.4 (0.1 to 0.6)
days per eligible person	0.7 (0.3 to 1.2)	0.1 (−0.1 to 0.3)	0.2 (0 to 0.5)	0.3 (0.1 to 0.5)	0.4 (0.1 to 0.7)
days per person screened at least once	0.8 (0.4, 1.2)	0.1 (−0.1 to 0.3)	0.3 (0 to 0.5)	0.4 (0.1 to 0.6)	0.4 (0.1 to 0.8)
days per health check	0.0 (−0.2 to 0.2)	0.0 (−0.1 to 0.1)	0.0 (−0.1 to 0.1)	0.1 (0 to 0.2)	0.0 (−0.1 to 0.1)
days per head (most deprived quintile group)	0.7 (−0.3 to 1.7)	0.7 (−0.3 to 1.7)	0.2 (−0.3 to 0.9)	0.4 (−0.2 to 1)	0.3 (−0.4 to 1.2)
days per head (least deprived quintile group)	0.6 (0 to 1.2)	0.0 (0.0 to 0.0)	0.1 (−0.1 to 0.5)	0.3 (−0.1 to 0.7)	0.4 (−0.2 to 0.9)
**Additional life gained over the 60 years of follow-up**					
Total (years for whole population)	1,510 (574 to 2,540)	242 (−208 to 626)	542 (−88.2 to 1,140)	716 (177 to 1,240)	868 (171 to 1,630)
days per head of population	0.6 (0.2 to 0.9)	0.1 (−0.1 to 0.2)	0.2 (0 to 0.4)	0.3 (0.1 to 0.5)	0.3 (0.1 to 0.6)
days per eligible person	0.6 (0.2 to 1.1)	0.1 (−0.1 to 0.3)	0.2 (0 to 0.5)	0.3 (0.1 to 0.5)	0.4 (0.1 to 0.7)
days per person screened at least once	0.7 (0.3 to 1.1)	0.1 (−0.1 to 0.3)	0.2 (0 to 0.5)	0.3 (0.1 to 0.6)	0.4 (0.1 to 0.7)
days per health check	0.0 (−0.2 to 0.2)	0.0 (−0.1 to 0.1)	0.0 (−0.1 to 0.1)	0.1 (0.0 to 0.2)	0 (−0.1 to 0.1)
days per head (most deprived quintile group)	0.6 (−0.5 to 1.7)	0.6 (−0.5 to 1.7)	0.2 (−0.3 to 0.9)	0.4 (−0.4 to 1.0)	0.3 (−0.5 to 1.2)
days per head (least deprived quintile group)	0.5 (−0.1 to 1.1)	0.0 (0.0 to 0.0)	0.1 (−0.1 to 0.5)	0.2 (−0.1 to 0.7)	0.3 (−0.2 to 0.9)

Abbreviations: CVD, cardiovascular disease; HC, an NHS Health Check; IHD, ischaemic heart disease; QALY, quality-adjusted life year. Deprivation quintile groups are based on the Index of Multiple Deprivation score for the area of residence. Health outcomes (cases prevented, premature deaths prevented, and days of quality-adjusted life and days of life gained) are expressed relative to the existing programme. Cases prevented are all cases prevented when following a cohort of 1 million adults aged 40–45 years until either 80 or 100 years of age. Premature deaths prevented are all-cause deaths prevented before age 75 or 80 years, when following a cohort of 1 million adults aged 40–45 years until 80 years of age. Additional quality-adjusted life gained and additional life gained are over the 60 years of follow-up, i.e., the remaining lifetime of the cohort. Estimates of 95% credible intervals (due to parameter uncertainty) are shown in parentheses. The 95% credible intervals are the 2.5th and 97.5th percentile of all estimates from 100 simulations.

^±^ High risk of CVD was defined as QRisk2 > 20%.

Increasing uptake by 30% amongst those living in the most deprived areas (bottom fifth) is an effective means to reduce inequalities, although it is associated with relatively small gains in measures of average population health.

### Increasing treatment

A 2.5-fold increase in the likelihood of starting treatment amongst those eligible was associated with relatively large improvement in indices of population health (Table 6) compared to increases in attendance or changes in eligibility criteria. The largest gains are seen for a 2.5-fold increase in statin treatment. Increasing all treatments 2.5-fold increases the health benefits of the programme 2- to 3-fold (950 deaths versus 390; 3,400 people free of 1 of the 4 diseases versus 1,370; 25,000 additional QALYs versus 10,000; 22,000 additional years of life versus 9,000). Increasing treatment rates is associated with compression of morbidity (the increase in QALYs is greater than the increase in survival).

**Table 6:** Effect of increasing treatment on process and outcomes measures of the National Health Service (NHS) health check programme (*n* = 1,000,000).

	2.5-fold increase in the likelihood of starting treatment at assessment amongst eligible patients				
	Statins	Antihypertensives	Smoking cessation services	Weight loss programme	All treatments
**Eligibility and uptake**					
Eligible for HC at any time (%)	85.1	85.1	85.1	85.1	85.1
Have one or more HCs (%)	79.6	79.6	79.6	79.6	79.6
Mean HCs per head of population	1.9	1.9	1.9	1.9	1.9
**Treatment (proportion of whole population)**					
Eligible for HC and any treatment (%)	81.0	81.0	81.0	81.0	81.0
Attended HC and were eligible for any treatment when attending (%)	73.1	73.2	73.2	73.2	73.1
Offered any treatment through HC (%)	34.2	29.4	27.5	39.7	47.4
Offered statins through HC (%)	19.5	8.4	8.4	8.4	19.5
Offered antihypertensives through HC (%)	3.3	8.1	3.3	3.3	8.0
Referred to a weight loss programme through HC (%)	17.6	17.6	17.6	33.3	33.3
Referred to smoking cessation services through HC (%)	1.2	1.2	2.9	1.2	2.9
Treated with statins through HC through HC (%)	10.3	4.3	4.3	4.3	10.3
Treated with antihypertensives through HC (%)	1.8	4.5	1.8	1.8	4.5
Attended a weight loss programme after HC referral (%)	9.9	9.9	9.9	21.2	21.2
Attended smoking cessation services after HC referral (%)	0.1	0.1	0.3	0.1	0.3
**Additional cases prevented by age 80**					
IHD	1,493 (1,084 to 1,910)	67 (28 to 110)	16 (-17 to 48)	14 (0 to 31)	1,589 (1,184 to 2,020)
Stroke	698 (484 to 911)	37 (9 to 67)	10 (−10 to 39)	8 (−4 to 25)	753 (528 to 979)
Dementia	53 (9 to 98)	77 (43 to 123)	−3 (−17 to 10)	76 (38 to 123)	202 (132 to 281)
Lung cancer	0 (−5 to 0)	0 (0 to 0)	127 (79 to 182)	0 (0 to 0)	127 (77 to 182)
Additional people living free of one of our diseases at age 80 years	1,697 (1,255 to 2,147)	124 (72 to 179)	108 (58 to 162)	67 (26 to 107)	1,995 (1,550 to 2,440)
**Additional cases prevented (by age 100)**					
IHD	1,825 (1,254 to 2,486)	52 (10 to 94)	1 (−37 to 42)	1 (−20 to 17)	1,878 (1,292 to 2,538)
Stroke	921 (613 to 1,264)	31 (1 to 63)	−2 (−31 to 29)	3 (−18 to 23)	952 (647 to 1,309)
Dementia	−20 (−84 to 49)	109 (52 to 174)	−22 (−44 to 1)	129 (57 to 208)	195 (65 to 316)
Lung cancer	0 (−5 to 0)	0 (0 to 0)	255 (161 to 366)	0 (0 to 0)	255 (159 to 366)
Additional people living free of one of our diseases at age 100 years	1,503 (1,049 to 2,073)	96 (54 to 147)	120 (49 to 176)	66 (24 to 119)	1,787 (1,334 to 2,368)
**Additional deaths prevented (all cause) over the 60 years of follow-up**					
<75 years	265 (191 to 363)	23 (3 to 50)	64 (26 to 106)	12 (0 to 35)	364 (276 to 474)
<80 years	423 (300 to 553)	37 (15 to 75)	79 (30 to 128)	23 (4 to 50)	563 (423 to 731)
**Additional quality-adjusted life gained over the 60 years of follow-up**					
Total (QALYs for whole population)	11,600 (8,260 to 15,000)	1,120 (704 to 1,690)	1,730 (840 to 2,660)	782 (324 to 1,330)	15,200 (11,700 to 19,030)
days per head of population	4.2 (3 to 5.5)	0.4 (0.3 to 0.6)	0.6 (0.3 to 1.0)	0.3 (0.1 to 0.5)	5.6 (4.3 to 7)
days per eligible person	4.7 (3.4 to 6.1)	0.5 (0.3 to 0.7)	0.7 (0.4 to 1.1)	0.3 (0.1 to 0.5)	6.3 (4.9 to 7.9)
days per person screened at least once	5.3 (3.8 to 6.8)	0.5 (0.3 to 0.8)	0.8 (0.4 to 1.2)	0.4 (0.1 to 0.6)	7 (5.5 to 8.7)
days per health check	2.2 (1.6 to 2.9)	0.2 (0.1 to 0.3)	0.3 (0.2 to 0.5)	0.2 (0.1 to 0.3)	2.9 (2.3 to 3.6)
days per head (most deprived quintile group)	6.0 (3.8 to 8.6)	0.5 (0.1 to 1)	0.6 (−0.1 to 1.5)	0.4 (0.0 to 0.9)	7.5 (5.1 to 10.4)
days per head (least deprived quintile group)	3.8 (2.5 to 5.6)	0.4 (0.2 to 0.8)	0.5 (−0.1 to 1.1)	0.3 (0.0 to 0.6)	4.9 (3.5 to 6.9)
**Additional life gained over the 60 years of follow-up**					
Total (years for whole population)	9,970 (7,080 to 13,400)	913 (512 to 1,420)	1,750 (855 to 2,780)	632 (229 to 1,120)	13,300 (10,300 to 16,800)
days per head of population	3.6 (2.6 to 4.9)	0.3 (0.2 to 0.5)	0.6 (0.3 to 1.0)	0.2 (0.1 to 0.4)	4.8 (3.8 to 6.1)
days per eligible person	4.1 (2.9 to 5.4)	0.4 (0.2 to 0.6)	0.7 (0.4 to 1.1)	0.3 (0.1 to 0.4)	5.5 (4.3 to 6.9)
days per person screened at least once	4.6 (3.3 to 6.0)	0.4 (0.2 to 0.7)	0.8 (0.4 to 1.3)	0.3 (0.1 to 0.5)	6.1 (4.8 to 7.6)
days per health check	1.9 (1.4 to 2.5)	0.2 (0.1 to 0.3)	0.3 (0.2 to 0.5)	0.1 (0.0 to 0.2)	2.6 (2.0 to 3.2)
days per head (most deprived quintile group)	5.2 (3.3 to 7.9)	0.4 (0.1 to 0.9)	0.6 (−0.2 to 1.5)	0.3 (0.0 to 0.8)	6.5 (4.2 to 9.6)
days per head (least deprived quintile group)	3.3 (2.1 to 5.0)	0.3 (0.1 to 0.7)	0.5 (−0.1 to 1.1)	0.2 (0 to 0.5)	4.3 (2.8 to 6.1)

Abbreviations: HC, an NHS Health Check; IHD, ischaemic heart disease; QALY, quality-adjusted life year. Deprivation quintile groups are based on the Index of Multiple Deprivation score for the area of residence. Health outcomes (cases prevented, premature deaths prevented, days of quality-adjusted life, and days of life gained) are expressed relative to the existing programme. Cases prevented are all cases prevented when following a cohort of 1 million adults aged 40–45 years until either 80 or 100 years of age. Premature deaths prevented are all-cause deaths prevented before age 75 or 80 years, when following a cohort of 1 million adults aged 40–45 years until 80 years of age. Additional quality-adjusted life gained and additional life gained are over the 60 years of follow-up, i.e., the remaining lifetime of the cohort. Estimates of 95% credible intervals (due to parameter uncertainty) are shown in parentheses. The 95% credible intervals are the 2.5th and 97.5th percentile of all estimates from 100 simulations.

A strategy that combines extending eligibility to those with preexisting hypertension, extending the upper age of eligibility to 79 years, increasing uptake by 30%, and increasing treatment rates 2.5-fold among eligible patients (i.e., ‘maximum potential’ scenario) results in at least a 3-fold increase in benefits compared to no programme (1,360 premature deaths versus 390; 5,100 people free of 1 of the 4 diseases versus 1,370; 37,000 additional QALYs versus 10,000; 33,000 additional years of life versus 9,000).

### Effect on health equity

The current programme has a greater absolute impact on health for those living in the most deprived areas compared to those living in the least deprived areas (gain in quality-adjusted life of 5.1 days for those in the most deprived area versus 3.3 days for those in the least deprived area; gain in life expectancy of 4.4 days versus 2.8 days, respectively).

**Fig 2:**
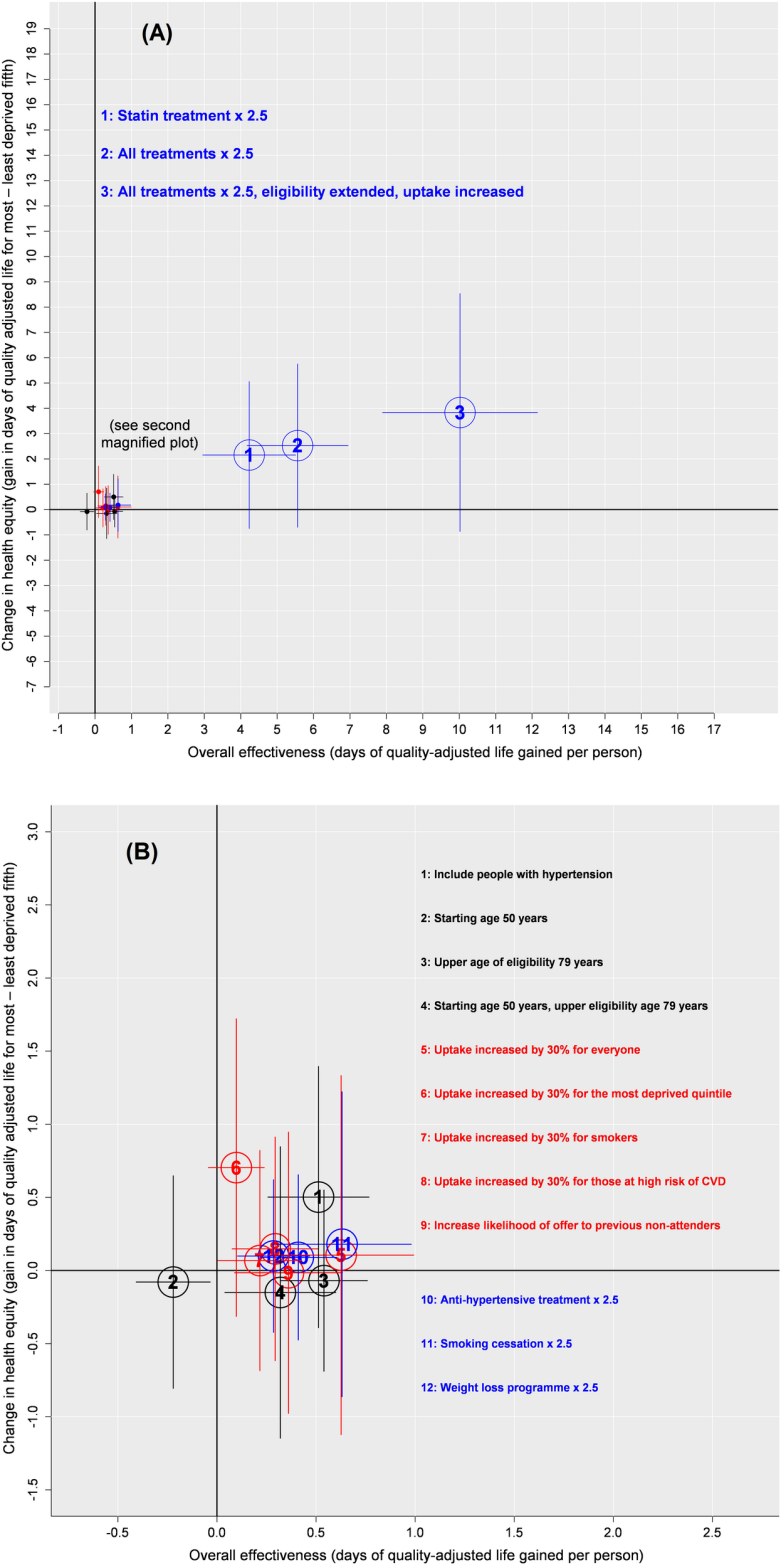
Health equity plot showing the effect of modifications to the existing programme on overall effectiveness (health gain) and on health equity.

We summarise our estimates on health equity and overall effectiveness in Fig 2. Most modifications to the programme that are associated with an improvement in health equity are also associated with an improvement in overall population health.

### Value of information analyses

A value of information analysis that considered the different sources of parametric uncertainty captured within the model (Table A in S1 Data and Table B in S1 Data) showed that the parameters contributing most to the uncertainty in the results were the initial adherence to statin prescription (23% of variance) and the annual dropout rate from statins (15% of variance), both for analyses estimating the overall contribution of the current NHS Health Check programme and analyses estimating the benefit of the ‘maximum potential’ scenario.

### Sensitivity analyses

Assuming that past attendance predicted future attendance (i.e., those who previously attended were more likely to attend in the future) resulted in a smaller proportion of the population attending for one or more health checks (75% versus 80%), fewer health checks (1.6 versus 1.9 per head of population), and a marginally smaller overall improvement in population health (340 versus 390 premature deaths avoided; 1,200 versus 1,400 people living free of the 4 diseases at age 80 years). Under this assumption, the added benefit from a strategy of increasing likelihood of invitation to ‘non-attenders’ was similar (40 deaths versus 40; 130 people free of 1 of the 4 diseases at age 80 years versus 120; 940 additional QALYs versus 990; and 800 additional years of life versus 870).

Assuming that recent trends in incidence and case fatality for stroke and IHD continue would reduce the estimate of benefit of the current NHS Health Checks programme by around half (170 deaths versus 390; 890 people free of 1 of the 4 diseases versus 1,370; 5,700 additional QALYs versus 10,000; 4,600 additional years of life versus 9,000; Table 7). The estimate of ‘maximum potential’ (relative to present performance) also halved under the same assumption (400 deaths versus 970; 2,300 people free of 1 of the 4 diseases versus 3,700; 14,000 additional QALYs versus 27,000; 11,000 additional years of life versus 24,000; Table 7).

**Table 7:** Sensitivity analysis showing the impact of a reduction in cardiovascular disease (CVD) risk (incidence and case fatality) on the estimate of benefits attributable to the National Health Service (NHS) health check programme.

	Current NHS Health Check programme (compared to no programme)		‘Maximum potential’ scenario (compared to current programme)	
	No decline in CVD risk	Decline in CVD risk	No decline in CVD risk	Decline in CVD risk
**Additional cases prevented by age 80 (per million)**				
IHD	1,089 (817 to 1,367)	592 (465 to 794)	2,802 (2,236 to 3,459)	1,533 (1,263 to 1,915)
Stroke	525 (414 to 671)	269 (181 to 371)	1,335 (1,063 to 1,656)	704 (531 to 933)
Dementia	135 (72 to 190)	126 (53 to 208)	320 (220 to 462)	299 (155 to 447)
Lung cancer	90 (36 to 147)	94 (44 to 151)	207 (133 to 293)	221 (145 to 310)
**Additional people living free of one of our diseases at age 80 years**	1,371 (1,101 to 1,685)	886 (697 to 1,122)	3,562 (2,903 to 4,253)	2,286 (1,891 to 2,716)
Additional cases prevented by age 100 (per million)				
IHD	1,296 (898 to 1,730)	811 (580 to 1,129)	3,837 (2,833 to 4,962)	2,407 (1,840 to 3,009)
Stroke	679 (494 to 958)	400 (269 to 564)	1,993 (1,518 to 2,565)	1,215 (904 to 1,619)
Dementia	125 (32 to 222)	267 (141 to 393)	235 (75 to 384)	625 (421 to 853)
Lung cancer	175 (103 to 259)	186 (113 to 281)	435 (311 to 567)	469 (331 to 596)
Additional people living free of one of our diseases at age 100 years	1,235 (956 to 1,566)	1,044 (784 to 1,379)	3,654 (2,842 to 4,591)	3,007 (2,400 to 3,721)
**Additional premature deaths prevented (per million)**				
<75 years	246 (182 to 331)	101 (47 to 164)	544 (424 to 713)	211 (137 to 294)
<80 years	386 (291 to 499)	167 (82 to 253)	967 (777 to 1,215)	402 (287 to 516)
**Additional quality-adjusted life gained over the 60 years of follow-up**				
Total (QALYs for whole population)	10,300 (8,170 to 12,900)	5,700 (4,180 to 7,730)	27,400 (22,400 to 33,500)	14,400 (11,700 to 17,600)
days per head of population	3.8 (3.0 to 4.7)	2.1 (1.5 to 2.8)	10.0 (8.2 to 12.2)	5.2 (4.3 to 6.4)
days per eligible person	4.3 (3.4 to 5.4)	2.5 (1.8 to 3.3)	10.6 (8.7 to 12.9)	5.7 (4.7 to 6.9)
days per person screened at least once	4.7 (3.8 to 6.0)	2.7 (2.1 to 3.5)	11.3 (9.3 to 13.7)	6.0 (5.0 to 7.3)
days per health check	2.0 (1.6 to 2.5)	1.1 (0.8 to 1.4)	3.0 (2.5 to 3.8)	1.5 (1.2 to 1.9)
days per head (most deprived quintile group)	5.1 (3.4 to 7.1)	2.7 (1.2 to 4.2)	12.9 (9.0 to 16.7)	6.9 (4.7 to 9.7)
days per head (least deprived quintile group)	3.3 (2.1 to 4.5)	1.7 (0.7 to 2.8)	9.0 (6.6 to 11.3)	4.5 (2.7 to 6.4)
**Additional life gained over the 60 years of follow-up**				
Total (years for whole population)	9,700 (6,880 to 11,300)	4,620 (3,120 to 6,450)	24,000 (19,400 to 29,200)	11,500 (9,360 to 14,200)
days per head of population	3.3 (2.5 to 4.1)	1.7 (1.1 to 2.4)	8.8 (7.1 to 10.7)	4.2 (3.4 to 5.2)
days per eligible person	3.7 (2.8 to 4.8)	2.0 (1.4 to 2.7)	9.3 (7.6 to 11.3)	4.6 (3.7 to 5.6)
days per person screened at least once	4.1 (3.2 to 5.2)	2.2 (1.6 to 2.9)	9.9 (8.1 to 12)	4.8 (3.9 to 5.9)
days per health check	1.7 (1.4 to 2.2)	0.9 (0.6 to 1.2)	2.7 (2.1 to 3.3)	1.2 (0.9 to 1.5)
days per head (most deprived quintile group)	4.4 (2.7 to 6.5)	2.1 (0.7 to 3.6)	11.3 (7.6 to 15.3)	5.5 (3.3 to 7.8)
days per head (least deprived quintile group)	2.8 (1.7 to 4.0)	1.4 (0.4 to 2.5)	7.8 (5.5 to 9.9)	3.6 (1.9 to 5.5)

Abbreviations: HC, an NHS Health Check; IHD, ischaemic heart disease; QALY, quality-adjusted life year. Deprivation quintile groups are based on the Index of Multiple Deprivation score for the area of residence. Health outcomes (cases prevented, premature deaths prevented, days of quality-adjusted life, and days of life gained) are expressed relative to the existing programme. Cases prevented are all cases prevented when following a cohort of 1 million adults aged 40–45 years until either 80 or 100 years of age. Premature deaths prevented are all-cause deaths prevented before age 75 or 80 years, when following a cohort of 1 million adults aged 40–45 years until 80 years of age. Additional quality-adjusted life gained and additional life gained are over the 60 years of follow-up, i.e., the remaining lifetime of the cohort. Estimates of 95% credible intervals (due to parameter uncertainty) are shown in parentheses. The 95% credible intervals are the 2.5th and 97.5th percentile of all estimates from 100 simulations. The ‘maximum potential’ scenario models the effect of simultaneously widening eligibility to include those with a diagnosis of hypertension, increasing attendance by 30% for everyone and increasing all treatments by 2.5-fold.

Assuming considerable uncertainty in baseline CVD risk (multiplying the QRisk2 score by a value chosen from a log-normal distribution with 95% quantiles of 0.8 and 1.2) does not change the conclusion, although the 95% CrIs widen (approximately doubling; Table C in S1 Data).

## Discussion

### Principal findings

We estimate the current NHS Health Check programme is preventing approximately 300 premature deaths per year and resulting in 1,000 additional people aged 80 years being free of CVD, dementia, and lung cancer each year in England. It is increasing quality-adjusted life by around 4 days and life expectancy by around 3 days per person aged 40–45 years. There is potential to considerably improve the benefits of NHS Health Checks, notably by increasing uptake of treatments, increasing attendance, and extending the programme to include those with diagnosed hypertension. In keeping with the benefits of the current programme, most of these modifications are associated with an improvement in health equity and compression of morbidity.

### Strengths and limitations

Our work has several strengths. We have estimated the additional benefit of the NHS Health Check programme, over and above routine care, by using data that reflect changes in risk factors over time considering changes due to routine healthcare and by using estimates of additional treatments attributable to an NHS Health Check. By comparing NHS Health Checks with a counterfactual situation of no NHS Health Checks on the same population, we have eliminated the selection bias (i.e., healthy people who are concerned about their health choosing to attend a health check) that may affect observational studies of the programme. We have also made allowance for nonadherence and relapse of treatment. As a result, the model effectively simulates the impact of treatment decisions dissipating over time, resulting in a smaller estimate of benefit compared to approaches assuming fixed adherence over time. The microsimulation approach gives substantial flexibility, allowing exploration of different scenarios, testing of assumptions, and description of outcomes by subgroup.

By modelling differences in uptake by deprivation and disease incidence by deprivation (due to differences in risk factor prevalence and using QRisk2 score, which includes deprivation and ethnicity as predictive factors), we have been able to model health impacts by deprivation. However, we have not made allowance for likely differences in survival (case fatality) by deprivation, which may have led to an underestimation of the programme’s impact on reducing health inequalities [70,71]. We have also assumed the same level of adherence to treatment by deprivation. Different levels of adherence by deprivation might also affect the programme’s impact on inequalities.

We have represented uncertainty around multiple input parameters. Nonetheless, as with all models, there are parts for which we could find limited data (e.g., likelihood of repeat attendance for an NHS Health Check, attendance at a weight management programme after referral, and long-term compliance with medication), and the measured ‘parametric’ uncertainty may not capture the ‘true’ uncertainty in some of the parameters (e.g., disutility weights). We have not made any allowance for migration, although we note that inward migration is less common in the age group (over 40 years) that we studied [72].

We have also assumed that the present background incidence of disease continues and that no major new treatments will be developed. Different assumptions about future disease incidence or risk factors would alter the estimates of overall health benefit and might affect the overall estimate of relative benefit of the different scenarios. It is noticeable that the estimate of overall benefit falls by as much as half if the present downward trend in CVD continues for another 20 years. This underscores the challenges in making forecasts about the benefits in health attributable to the programme in the long term.

There are also differences in disease incidence and variation in programme delivery between local authorities, which may result in different estimates of absolute benefit, at a local level, to those presented here. Whilst dementia risk is modifiable [29,41], there is no trial evidence to demonstrate the effects of lipid-lowering therapy, weight loss, or smoking on dementia incidence, and consequently, the effects on dementia should be treated with caution.

There are aspects of the programme we have not considered. We have not estimated costs, nor have we considered harms of treatments. Notably, we have not considered the additional risk of type 2 diabetes attributable to statins, which might influence the modelled benefits of statins used in primary prevention [73,74]. Other side effects are likely to be short-lived, and thus, their overall contribution to (lifelong) disutility would be small, and allowance has been made for patients not starting and discontinuing medication (which may be a response to side effects and thus limit the duration of side effects). Elements of the programme we have not modelled include brief interventions around alcohol, benefits from early diagnosis (e.g., chronic kidney disease and type 2 diabetes). With the exception of lung cancer, other (noncardiovascular) health benefits (e.g., weight loss on cancer and smoking on other cancers and chronic obstructive pulmonary disease [COPD]) have not been modelled. In some settings, the largest contributor to QALY benefit from smoking cessation is through reduction in COPD [75]. Given this and the exclusion of other health outcomes, our estimate of health gain is likely to underestimate the overall health gain from the programme. We focused on the cardiovascular elements of the NHS Health Check because this was the original focus of the programme, because of the large burden of disease attributable to CVD, and because of the well-developed evidence base describing treatment uptake and effectiveness.

We note that the cardiovascular benefits attributable to weight loss interventions (Table 6) are less than for other treatments, despite a large number of referrals to weight management. This is in part because it was assumed that any weight loss was fully regained over a 5-year period. It may in part reflect the cardiovascular benefits of weight being modelled through QRisk2 score, which includes BMI. We did not additionally explicitly model the impact of BMI on other risk factors (e.g., cholesterol and blood pressure), as changes in these risk factors through changes in BMI have been implicitly modelled through the matching process that models change in risk factors over time (see Section 2.1 in S1 Text). Nonetheless, this approach may have underestimated the benefits of weight loss on CVD.

We have primarily considered benefits at the population level, which are different to benefits at the individual level. Stopping smoking is highly beneficial for health at an individual level, but that component of the programme is currently delivering limited population benefit because few individuals are being referred to smoking cessation services (through the NHS Health Check programme). This may reflect national declines in demand for smoking cessation services, in part due to declines in smoking prevalence and in part due to the availability of e-cigarettes. The individual benefit for an individual who is referred to smoking cessation services and who quits will be substantial. Whilst we have tried to choose scenarios based on what may be realistically or ideally attainable, we do not know what is possible. We note others have commented on the suboptimal uptake of treatments amongst people attending a health check [14]. Low uptake of treatments may reflect variation in quality of care but may be due to clinical factors (e.g., patient choice, side effects, or contraindications).[76]

This work concerns one aspect of secondary prevention of CVD. In terms of reducing morbidity and mortality from CVD other aspects of prevention, such as population-level approaches (e.g., cigarette taxation and reformulation to reduce salt) [75,77,78], and aspects of clinical care (e.g., management of acute coronary syndromes) are valuable. This work does not attempt to consider the relative contributions of these different elements, which others have done [15]. In practice, countries are likely to adopt a spectrum of approaches from primary prevention, at the population level, to the delivery of high-quality clinical care to individuals to reduce the burden of CVD [79].

### Model validation

There are different views on the appropriateness and meaning of the concept of validity in model development. We take the position that validity needs to be considered in the context of use. It is not possible for a model to be universally valid. Models can be used to answer multiple questions, and validity should be considered specifically for each question. Validation through comparing a model’s findings with empirical research is usually only partially possible, as models are typically used to answer questions that no single empirical study can answer. Other factors need to be considered when deciding whether a model is ‘valid’ or robust. We think our model is robust because of (1) the use of good data sources, e.g., national data on disease epidemiology, published data on programme performance, and estimates of treatment efficacy from trials; (2) the steps taken during model development, e.g., calibration and use of expert advice; (3) the comparisons of some model outputs with mortality data, i.e., effectively validating the population health module; (4) extensive stochastic uncertainty analysis and sensitivity analysis; and (5) comparisons with other modelling studies (described below). The uncertainty and sensitivity analyses suggest that the pattern of findings and the estimates of order of magnitude are broadly similar under a range of different assumptions for the comparisons of most interest to us. Substantial changes in CVD risk, now or in the future, and statin adherence have been identified as factors most likely to have a marked impact on estimate of benefit.

### Comparison to other work

We are not aware of any other work that has sought to estimate the impact of making changes to the NHS Health Check programme. We note that the original modelling work, undertaken by the Department of Health, suggested that 40 was the optimal age to start screening [80], and consistent with this, we found a small health loss associated with increasing the starting age to 50 years.

The original modelling undertaken by the Department of Health estimated that the programme could prevent 1,600 heart attacks and strokes and at least 650 premature deaths each year [81]. Kypridemos et al. estimated that a ‘vascular check programme’ in the UK might prevent approximately 1,000 nonfatal and 200 fatal cases of CVD annually [15]. While there are important differences between the models (e.g., effectiveness of statins and trends in CVD incidence), we note that despite this, these estimates and our own (1,700 events, of which 1,400 are attributable to CVD, and 300 premature deaths prevented per annum) are relatively similar. Our estimates for the increase in QALYs (3.8 days per head of population) are of a similar order of magnitude but are less than half of the estimates of a ‘health check’ programme (8.6 days per head of population) when following a population for 30 years using the Archimedes model, which has been validated for diabetes treatments [16,82]. While we cannot be sure how ‘valid’ these other model estimates are, if they are ‘valid’, these comparisons provide some reassurance concerning the validity of our model’s output.

Our work can also be compared to observational reports of the current programme. One study estimated the health benefits for those who attend for a health check compared to a matched control group who do not attend [83]. The paper reported similar levels of treatment to those that we modelled and reported changes in cardiovascular risk factors (e.g., 2.5 mmHg reduction in blood pressure) for attenders relative to nonattenders. We note that these differences are relatively large (our respective estimates being 0.07 mmHg for those who attend for a health check), at a population-level, and would result in much greater health improvements than those that we estimated. These discrepancies in health outcomes may be explained by residual confounding of the observational data (i.e., those who attend for a health check are healthier than those who do not) or may suggest other pathways through which the health check may be influencing cardiovascular risk, such as stimulating participants to adopt healthier habits (e.g., being physically active or eating a healthier diet).

Comparisons with other population-level interventions should be interpreted cautiously, as methods may vary and the nature and scale of the interventions can be very different. Structural interventions (e.g., taxing unhealthy foods, 2,300 CVD deaths averted; salt reformulation, 6,000 CVD deaths averted per annum) may have a greater impact on CVD [78,84]. Such interventions can be politically difficult to implement, and individual interventions may be better seen as a complement to structural approaches [79]. Comparisons with screening programmes may be instructive, as the programme shares some characteristics with screening programmes. Breast cancer screening in the UK is estimated to increase life expectancy by 6.9 days and QALYs by 2.0 days per eligible women [85], whilst bowel cancer screening is estimated to increase life expectancy by 4.6 to 9.6 days (depending on the screening model adopted) and QALYs by 3.8 to 10.2 days per eligible person [86]. Currently, the NHS Health Check programme is performing at the lower end of these estimates, although our work suggests it has the potential to exceed the benefits of these screening programmes.

### Interpretation and implications

Broadly, our work suggests the programme is contributing to benefits in population health and is also serving to reduce health inequalities. As the gain in time lived in full health is greater than the increase in survival, the programme is adding more good quality life years than it is adding years to life (i.e., it is compressing morbidity).

We have presented 2 contrasting metrics of benefit, one based around events avoided and a second based on increase in life expectancy (including QALYs). QALYs are important for making comparisons with other health interventions and making judgements about cost-effectiveness. We have presented numbers for the population, as a whole, and per head of population. The latter is standardised for the population size and is a common metric used to describe the benefit of other similar programmes, i.e., screening programmes. However, the mean benefit per person can be misleading, as the benefits are not evenly distributed. The events avoided may be a better way to communicate how the programme may offer a substantial benefit (prevention of major disease or premature death) for a small proportion of the population.

If the programme were optimised, the health benefits of the programme could be even greater. Our work suggests a number of approaches that might enable this. Increasing the uptake of treatments, principally statins, through the programme may be a more important strategy to increase the health benefits of the programme. This appears to be more important than increasing programme attendance, which has been a focus of efforts to improve the programme to date. It might be a more efficient use of resources to ensure the programme is optimally managing patients before seeking to increase attendance.

The benefits of statins offered through the programme were particularly pronounced. Given the recent recommendations from the UK’s National Institute for Health and Care Excellence (NICE) to lower the threshold for initiating statin therapy for primary prevention [87], it seems likely that there will be scope to increase statin therapy. Other interventions are estimated to have a smaller impact. Nonetheless, there is scope for their impact to be greater. For example, smoking cessation is highly beneficial but had a very low uptake [88]; the benefit of this component of the programme could be greater if more smokers were referred. It seems likely that weight loss interventions would be more beneficial if the weight loss was maintained. Overall, there may be a need for quality assurance around elements of the programme, as there are in screening programmes, to enable the programme to maximise its potential.

National data suggest some targeting of high-risk groups (e.g., based on deprivation or ethnicity) already occurs [15,44], and this approach has appeared to be successful in reducing health inequalities. Now that the programme is established, targeting those who did not attend in the previous 5-year cycle would also appear to be a sensible strategy, as a complement to existing targeting strategies. Given the relatively large incremental gain for extending the programme to include those with an existing diagnosis of hypertension, this change in eligibility criteria merits further exploration.

Given that we have not considered costs, it would be inappropriate to draw conclusions about the overall cost-effectiveness of the overall programme or the marginal cost-effectiveness of the change scenarios we explore. However, we note our estimates of benefits in terms of new cases of CVD prevented are similar to the estimates from the original modelling prior to programme’s introduction, which suggested that the programme was cost-effective [80].

### Future research

We have primarily considered health benefits. Whilst a detailed assessment of health impact is important in making decisions about the NHS Health Check programme, future work should integrate cost data and undertake a cost-effectiveness or cost-utility analysis, which will provide a stronger basis for decision making. Future modelling work may want to consider other scenarios around changes in delivery, e.g., targeting known smokers or opportunistic health checks in primary care. Our scenarios on the potential increase in uptake of different treatment for statins are based on what is being achieved in one well-performing area. However, there is considerable uncertainty about what could be achieved (at scale) and how much it would cost. What our study does provide is an indication of how much additional health benefit could be realised if these scenarios were achieved. Further work might want to explore why referral and treatment rates are apparently low and how these rates might be increased. As data on the operation of the programme improve, researchers will be better able to estimate the current and potential future health impacts. Programme data should also allow direct observation of benefit (e.g., change in blood pressure), although in the absence of randomised controlled trials, issues concerning selection bias will limit the use of such comparisons. However, observational studies may lack power to detect the differences in ‘hard’ health outcomes at a population level that we report in this paper. Modelling will continue to be necessary to investigate longer-term outcomes of the current programme and to address ‘what if’ questions about the possible benefits of making changes to the programme. Other similar models do exist [15,16,73]. Formal comparisons between models may serve as a form of validation and means to identify the respective strengths and weaknesses of the different models.

### Summary

Our work suggests that the current NHS Health Check programme is contributing to improvements in population health, a narrowing of health inequalities, and compressing morbidity. There appears to be considerable scope to improve the health benefit of the programme, particularly by ensuring those who are assessed and eligible for treatment receive appropriate treatment. Focusing on inviting previous nonattenders and widening the eligibility criteria to include those with an existing diagnosis of hypertension could also make a valuable contribution to increasing the health benefits of the programme.

## Supporting information

S1 Data Comparison of observed and modelled mortality for ischaemic heart disease and stroke. (DOCX)

S2 Data Results supplement. (DOCX)

S1 Text Methods supplement (technical appendix). (DOCX)

## Abbreviations

COPD: chronic obstructive pulmonary disease
CrI: credible interval
CVD: cardiovascular disease
ELSA: English Longitudinal Study of Aging
IHD: ischaemic heart disease
NHS: National Health Service
NICE: National Institute for Health and Care Excellence
QALY: quality-adjusted life year
