# Supplementary material for: The current and potential health benefits of the National Health Service Health Check cardiovascular disease prevention programme in England: A microsimulation study
Source: PLoS Med. 2018 Mar 6;15(3):e1002517. doi: 10.1371/journal.pmed.1002517 (PMC5839536; doi:10.1371/journal.pmed.1002517)
Supplement: S1 Data — (DOCX) [file pmed.1002517.s001.docx]

**Comparison of observed and modelled mortality for ischaemic heart disease and stroke**

Observed data indicated by horizontal bars for five year age bands for England & Wales based on certified cause of death (<https://www.ons.gov.uk/peoplepopulationandcommunity/birthsdeathsandmarriages/deaths/bulletins/deathsregisteredinenglandandwalesseriesdr/2012-11-06>); Modelled data indicated by continuous line and estimated for each year of life from aged 40 years; IHD = ischaemic heart disease.
