# Supplementary material for: The current and potential health benefits of the National Health Service Health Check cardiovascular disease prevention programme in England: A microsimulation study
Source: PLoS Med. 2018 Mar 6;15(3):e1002517. doi: 10.1371/journal.pmed.1002517 (PMC5839536; doi:10.1371/journal.pmed.1002517)
Supplement: S2 Data — (DOCX) [file pmed.1002517.s002.docx]

Table A. Value of Information analysis showing the change and percentage reduction in the standard deviation for the estimate of change in quality-adjusted life days attributable to the NHS Health Check Programme

| **Parameter** | **95% uncertainty interval in parameter** | **Source of uncertainty** | **Expected standard deviation if parameter known perfectly** | **Percentage of standard deviation attributable to uncertainty in the parameter** |
| --- | --- | --- | --- | --- |
| Initial rate of adherence to statins | 40% - 60% | Our assumption | 0.45 | 15% |
| Proportion of statin uses who stop each year | 3% - 7% | Our assumption | 0.46 | 13% |
| Proportion of those who are not eligible for a health check who receive one per year | 2% - 8% | Our assumption | 0.50 | 5% |

The estimated uncertainty (standard deviation) in the model output of interest if we were to learn the exact value of each uncertain parameter. The estimate of the change in quality adjusted life attributable to the NHS Health Check programme is 3.8 days (95% uncertainty interval 3.0 – 4.7, standard deviation 0.52). Only parameters that were estimated to explain 2% or more of the SD of the output are included in the table. Other parameters tested include relative uptake rates of an NHS Health Check between different groups of individuals, prescription rates of treatments, rates of adherence to treatments (other than statins), effectiveness of treatments, and parameters measuring relative quality of life in different health states.

Table B. Value of Information analysis showing the change and percentage reduction in the standard deviation for the estimate of additional increase in quality-adjusted life days attributable to the NHS Health Check Programme delivered under a ‘best case’ scenario compared to current performance

| **Parameter** | **95% uncertainty interval in parameter** | **Source of uncertainty** | **Expected standard deviation if parameter known perfectly** | **Percentage of standard deviation attributable to uncertainty in the parameter** |
| --- | --- | --- | --- | --- |
| Initial rate of adherence to statins | 40% - 60% | Our assumption | 0.87 | 23% |
| Proportion of statin uses who stop each year | 3% - 7% | Our assumption | 0.96 | 15% |

The estimated uncertainty (standard deviation) in the model output of interest if we were to learn the exact value of each uncertain parameter. The estimate of the change in quality adjusted life years attributable to the NHS Health Check programme delivered under a ‘best case’ scenario compared to current performance is 10.0 days (95% uncertainty interval 8.2-12.2 days, standard deviation 1.14)

Only parameters that were estimated to explain 2% or more of the SD of the output are included in the table. Other parameters tested include relative uptake rates of an NHS Health Check between different groups of individuals, prescription rates of treatments, rates of adherence to treatments (other than statins), effectiveness of treatments, and parameters measuring relative quality of life in different health states.

Table C. Sensitivity Analysis showing the impact of modelling uncertainty in baseline CVD risk on estimates of benefit of the NHS Health Check Programme based on current performance and on ‘maximal potential’ performance

|  | **Current NHS Health Check Programme (compared to no programme)** | | **‘Maximum potential’ scenario (compared to current programme)** | |
| --- | --- | --- | --- | --- |
|  | Standard model | Uncertainty in baseline CVD risk  Modelled^+^ | Standard model | Uncertainty in baseline CVD risk  modelled^+^ |
| **Additional cases prevented by age 80* (per million)** | | | | |
| IHD | 1089 (817, 1367) | 1130 (678, 1929) | 2802 (2236, 3459) | 2842 (1777, 4550) |
| Stroke | 525 (414, 671) | 543 (262, 975) | 1335 (1063, 1656) | 1350 (812, 2286) |
| Dementia | 135 (72, 190) | 104 (29, 200) | 320 (220, 462) | 255 (98, 418) |
| Lung Cancer | 90 (36, 147) | 89 (39, 149) | 207 (133, 293) | 213 (136, 303) |
| Additional people living free of one of our diseases at age 80 years | 1371 (1101, 1685) | 1403 (864, 2292) | 3562 (2903, 4253) | 3566 (2394, 5501) |
| **Additional cases prevented by age 100* (per million)** | | | | |
| IHD | 1296 (898, 1730) | 1339 (796, 2342) | 3837 (2833, 4962) | 3820 (2291, 6321) |
| Stroke | 679 (494, 958) | 694 (335, 1251) | 1993 (1518, 2565) | 2013 (1225, 3445) |
| Dementia | 125 (32, 222) | 155 (21, 301) | 235 (75, 384) | 339 (105, 575) |
| Lung Cancer | 175 (103, 259) | 174 (110, 244) | 435 (311, 567) | 444 (348, 585) |
| Additional people living free of one of our diseases at age 100 years | 1235 (956, 1566) | 1269 (810, 2098) | 3654 (2842, 4591) | 3694 (2611, 5658) |
| **Additional premature deaths prevented (per million)*** | | | | |
| <75 years | 246 (182, 331) | 229 (134, 367) | 544 (424, 713) | 497 (293, 779) |
| <80 years | 386 (291, 499) | 365 (219, 584) | 967 (777, 1215) | 900 (584, 1349) |
| **Additional days of quality-adjusted life gained* over the 60 years of follow-up** | | | | |
| Total (QALYs for whole population) | 10300  (8170, 12900) | 10200  (6410, 16400) | 27400  (22400, 33500) | 26500  (18400, 39500) |
| days per head of population | 3.8 (3.0, 4.7) | 3.7 (2.3, 6) | 10 (8.2, 12.2) | 9.7 (6.7, 14.4) |
| days per eligible person | 4.3 (3.4, 5.4) | 4.3 (2.7, 6.9) | 10.6 (8.7, 12.9) | 10.4 (7.3, 15.5) |
| days per person screened at least once | 4.7 (3.8, 6) | 4.8 (3, 7.7) | 11.3 (9.3, 13.7) | 11.1 (7.7, 16.4) |
| gays per health check | 2 (1.6, 2.5) | 2 (1.3, 3.1) | 3 (2.5, 3.8) | 2.9 (2, 4.3) |
| days per head (most deprived quintile group) | 5.1 (3.4, 7.1) | 5 (2.8, 8.5) | 12.9 (9, 16.7) | 12.4 (8.1, 19) |
| days per head (least deprived quintile group) | 3.3 (2.1, 4.5) | 3.2 (1.7, 5.3) | 9 (6.6, 11.3) | 8.7 (5.4, 13.8) |
| **Additional life gained* over the 60 years of follow-up** | | | | |
| Total (years for whole population) | 89700  (6880, 11300) | 8790  (5390, 14400) | 24000  (19400, 29200) | 22900  (15800, 33900) |
| days per head of population | 3.3 (2.5, 4.1) | 3.2 (2, 5.2) | 8.8 (7.1, 10.7) | 8.4 (5.8, 12.4) |
| days per eligible person | 3.7 (2.8, 4.8) | 3.7 (2.3, 6.1) | 9.3 (7.6, 11.3) | 9 (6.1, 13.3) |
| days per person screened at least once | 4.1 (3.2, 5.2) | 4.1 (2.6, 6.7) | 9.9 (8.1, 12) | 9.6 (6.6, 14.2) |
| gays per health check | 1.7 (1.4, 2.2) | 1.7 (1.1, 2.8) | 2.7 (2.1, 3.3) | 2.5 (1.7, 3.7) |
| days per head (most deprived quintile group) | 4.4 (2.7, 6.5) | 4.4 (2.4, 7.5) | 11.3 (7.6, 15.3) | 10.8 (6.7, 16.3) |
| days per head (least deprived quintile group) | 2.8 (1.7, 4.0) | 2.8 (1.4, 4.5) | 7.8 (5.5, 9.9) | 7.5 (4.4, 12) |

HC = an NHS Health Check; IHD = ischaemic heart disease; deprivation quintile groups are based on the Index of Multiple Deprivation score for the area of residence; *Health outcomes (cases prevented, premature deaths prevented, days of quality adjusted life and days of life gained) are expressed relative to the existing programme; Cases prevented are all cases prevented when following a cohort of 1 million adults aged 40-45 years until either 80 or 100 years of age. Premature deaths prevented are all-cause deaths prevented before age 75 or 80 years, when following a cohort of 1 million adults aged 40-45 years of age until 80 years of age. Standard errors shown in brackets; HC = health checks; additional days of quality adjusted life gained and additional days of life gained are over the 60 years of follow-up, i.e. the remaining lifetime of the cohort. ‘Maximum potential’ scenario models the effect of simultaneously widening eligibility to include those with a diagnosis of hypertension, increasing attendance by 30% for everyone and increasing all treatments by 2.5 fold; ^+^ explicitly models uncertainty in baseline CVD risk by multiplying the QRisk2 score by a value chosen from a log-normal distribution with 95% quantiles of 0.8 and 1.2.
