# Supplementary material for: The current and potential health benefits of the National Health Service Health Check cardiovascular disease prevention programme in England: A microsimulation study
Source: PLoS Med. 2018 Mar 6;15(3):e1002517. doi: 10.1371/journal.pmed.1002517 (PMC5839536; doi:10.1371/journal.pmed.1002517)
Supplement: S1 Text — (DOCX) [file pmed.1002517.s003.docx]

**Technical Appendix**

1. **General model overview**

The Health Check Microsimulation Model was developed to assess the health impact of interventions that seek to prevent cardiovascular disease and that are delivered through the NHS Health Check programme. A graphical outline of the model is given in Figure A. In short, the model consists of two functional parts:

(1) **A population health model** containing a population of individuals aged 40 – 45 years at baseline and which simulates the development of risk factors, disease incidence and mortality over time. This module used data from the Health Survey of England (HSE) and the English Longitudinal Study of Ageing (ELSA) to simulate risk factors for a synthetic population forward in time, based upon their baseline characteristics and likely changes in risk factors. This part of the health check model is further explained in following sections [2](#Ref451749296).

(2) **A model of the Health Check process** which simulated an eligibility assessment, likelihood of attendance, assessment for treatment and treatment effectiveness (including adherence to treatment). Briefly, based on eligibility criteria, some individuals in the population were eligible to attend a health check. Following an invitation, some of these individuals were assumed to attend. Those who attended had an assessment for eligibility for treatment based on cardiovascular risk factors. Those were eligible may be offered treatment. If the individual took up treatment through the Health Check programme, the corresponding risk factors were changed using estimated treatment effects, making allowance for adherence to treatment. The assumptions are further detailed in Section 3.

The model has an annual cycle, so that all quantities in the model were assumed to be constant within each year.


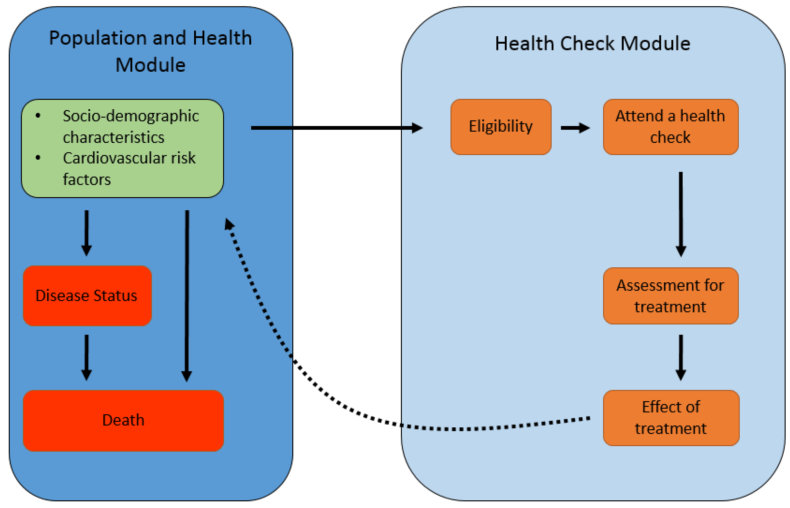


Figure A: Schematic overview of the NHS Health check model

1. **Population and health model**

Information to define synthetic individuals in the model at baseline was obtained from the Health Survey for England (HSE) 2009-2012. We only included individuals between 40 and 45 years and who had cholesterol, blood pressure and HbA1c recorded as part of a nurse visit separate to the main interview, a sub-sample of 3,247 individuals. The data at baseline are summarised in Table A.

Table A. HSE baseline data in the health check model

|  | **Mean or percentage** |
| --- | --- |
| Age (years) | 42.5 (IQR 41-44) |
| Systolic blood pressure (mmHg) | 121.5 (IQR 111.5-129.5) |
| Diastolic blood pressure (mmHg) | 75 (IQR 67.5-81.5) |
| Total cholesterol (mmol/L) | 5.4 (IQR 4.8-6) |
| HDL cholesterol (mmol/L) | 1.5 (IQR 1.2-1.7) |
| HbA1C (%) | 5.6 (IQR 5.3-5.7) |
| Male | 43.9% |
| Atrial fibrillation | 0% |
| Rheumatoid arthritis | 0% |
| Family history of cardiovascular disease | 0% |
| Chronic kidney disease | 0% |
| On blood pressure treatment | 5.1% |
| Index of multiple deprivation |  |
| 1 | 22.9% |
| 2 | 20.9% |
| 3 | 19.9% |
| 4 | 19.4% |
| 5 | 16.8% |
| Diabetes |  |
| None | 97.2% |
| Type 1 | 0.6% |
| Type 2 | 2.2% |
| Smoking |  |
| Never | 54.1% |
| Ex-smoker | 22.8% |
| Current <10 | 7.3% |
| Current 10-19 | 9.9% |
| Current 20+ | 5.9% |
| Education |  |
| ≥10 years or equivalent | 40.5% |
| 7-9 years | 49% |
| ≤6 years | 10.5% |
|  |  |

From this sub-sample of individuals, a synthetic population was drawn using stratified sampling with replacement. First, the proportion of the simulated population in each sex and ethnicity stratum was defined by 2011 census data (Table B). Then within each sex-ethnicity stratum, the simulated population was sampled at random from the equivalent subset of people from the equivalent stratum within the Health Survey of England sub-sample.

Table B- Cell percentages of individuals of different sex-ethnicity combinations in England in the 2011 census, among those age 40-45 years of age

|  | **White** | **Asian** | **Black** | **Mixed or other** |
| --- | --- | --- | --- | --- |
| **Women** | 5.06% | 0.63% | 0.28% | 0.19% |
| **Men** | 5.68% | 0.51% | 0.28% | 0.18% |

- 1. **Simulating risk factor trajectories by sampling from longitudinal data in ELSA**

After the baseline population, their socio-demographic characteristics and risk factors have been set, by sampling from the HSE data, the model proceeds by simulating the annual change in each variable for each simulated person.

Background trajectories for each variable, in the absence of health checks, were simulated using data from the English Longitudinal Study of Ageing (ELSA), covering years 1998 to 2012. This gave longitudinal data for all time-varying risk factors of interest in the model: blood pressure (both systolic and diastolic), HbA1c, body mass index (BMI), smoking status (in five categories), and cholesterol (both HDL and LDL).

During the simulation, at each annual time step, each individual in our simulated population was ‘matched’ to an individual in the ELSA dataset who had similar risk factors. The change in each risk factor for the synthetic individual in the next two to four years was then set to the corresponding change observed for the individual in ELSA, linearly adjusted for the difference in the time steps. This process was repeated until the end of simulation. This assumed a first-order Markov process where the next simulated value of a risk factor only depended on the current value and other predictors.

The details of this approach were different for each risk factor we simulated. We illustrate it for BMI.

1. The simulated individual was placed into one of 8 x 2 x 3 x 12 categories defined by the combination of age groups (8), gender (2), smoking status (3) and BMI category (12). The baseline measurement in year 0 is ‘BMI(0)’.
2. The ELSA dataset was restricted to observations where both BMI is observed and a value of BMI four years later was recorded for that same person.
3. Each such observation from ELSA was placed into one of these 8 x 2 x 3 x 12 categories.
4. If there exist any ELSA observations in the same category as the simulated individual, then a random one of these observations was drawn.
5. If there are no ELSA observations matching this category, then we coarsened the categories, using four age groups, three smoking groups and three BMI groups (i.e. 4 x 3 x 3), and rematched. This resulted in a match for every observation.
6. The next value of BMI for the randomly-chosen matching individual in ELSA will be four years later (which we will call BMI(4)).
7. The BMI values for the simulated individual in the next four years were defined as BMI(r) = BMI(0) + r x [BMI(4) – BMI(0)]/4, using linear interpolation, for r = 1, 2, 3, 4.
8. BMI values for the next set of four years, were estimated by returning to step (1), using BMI(4) and re-matching to ELSA data.

A different set of predictor variables was used to match simulated individuals to individuals in ELSA for each different variable we simulated, as listed in Table C. We used variables which were predictive of the next risk factor value for an individual and which always included the current risk factor value. Continuous predictors such as age and BMI were divided into categories, with a potentially different number of categories used for each outcome variable predicted. We used more categories for stronger predictor variables.

Table C: Variables used for matching synthetic individuals to individuals in ELSA

| **Variable to be simulated** | **Frequency of measurement in ELSA (years)** | **ELSA waves used** | **Predictor variables defining bins (with number of categories)** | | |
| --- | --- | --- | --- | --- | --- |
|  | | | **First match** | **Second match** | **Third match** |
| **Blood pressure** | 4 | 1,3,5,7 | Age (3), BMI(3), systolic blood pressure(11), diastolic blood pressure(6) | Age(3), BMI(3), systolic blood pressure (11) |  |
| **BMI** | 4 | 1,3,5,7 | Age(8), gender(2), smoking(3), BMI(12) | Age(4), smoking (3), BMI(3) |  |
| **Cholesterol and HDL** | 4 | 5,7 | Age(3), gender(2), BMI(3), income(3), cholesterol(8) | Gender(2), cholesterol  (8), BMI(3) | Gender(2), cholesterol(8) |
| **HbA1c and diabetes diagnosis** | 4 | 3,5,7 | Age(3), gender(2), BMI(3), HbA1C(6), income(2), diabetes(2) | BMI(3), HbA1C(6), diabetes(2) | HbA1C(6), diabetes(2) |
| **Smoking status** | 2 | 1, 2, 3, 4, 5, 6, 7 | Gender(2), age(4), BMI(3), smoking(5), income(3) |  |  |

For smoking status, no coarsening and re-matching was required, since the first categorization resulted in a match for all individuals in the HSE. For cholesterol and HbA1C, a third round of matching was required.

For smoking status, observations from ELSA were available at 2-yearly intervals, while the other outcomes we predicted were only available at 4-yearly intervals.

Hypertension diagnoses were simulated based on the simulated blood pressure data. A random 5% of people with systolic blood pressure of over 150mmHg were assumed to be diagnosed with hypertension each year.

Diabetes diagnoses were simulated based on diabetes diagnosis data in ELSA. Diabetes was also assumed to be diagnosed in 5% of individuals per year whose HbA1c level is above 6.5. Once a person was diagnosed with diabetes, we assumed they have diabetes for the rest of their life. If we predicted from ELSA that a person would be diagnosed with diabetes within a four year timeframe, the year of the diagnosis was chosen at random.

Section 3 discusses how the simulated risk factor trajectories may be modified by treatment given through the NHS Health Check programme. We assumed that the background trajectories, representing a ‘real-world’ population will account for people being given risk factor-modifying treatments outside the NHS Health Check programme.

##

## Correcting matching process for smoking

Compared to published data,[1–3] ELSA predicted a higher quitting rate for smokers and a lower relapse rate to return to smoking after quitting. This may be attributable to reporting bias in ELSA (i.e. people preferentially reporting the behaviour that is ‘socially approved’). We adjusted the simulated changes in smoking status by reducing the 10 year quit rate from the average 6.5% observed in ELSA to 5%, and increasing the 2-year relapse rate to 37%. Quitting was defined as a change from any of the smoking categories to not smoking, and relapse as a change from being an ex-smoker to a moderate smoker. We assumed people were ex-smokers if there are two consecutive records in ELSA of not smoking.

Figure B: Summary of risk factors in the simulated population, over time

**2.2 Disease prevalence, incidence and mortality**

At the beginning of the simulation, a proportion of the population were assumed to have cardiovascular disease (CVD), according to age- and sex-specific prevalence of IHD and stroke from the DISMOD output. Individuals with highest QRISK2 score at baseline were then assigned to have either IHD or stroke. We assumed that nobody had dementia or lung cancer at baseline, reflecting the very low prevalence of these conditions in people aged 40-45 years.

We used risk scores (QRISK2 and CAIDE) to estimate each individual’s disease risk based on individual-level risk factors.[4–6] The risk scores are used to estimate the probability of an incident event for each individual within the next 10 years for cardiovascular disease (using the QRISK2 score) and the next 20 years for dementia (using the CAIDE score).

Case fatality rates by age were estimated from routine data sources (summarised in Table D), using the DISMOD II v1.05 program [4]. This also provided a second source of information on incidence, which was combined with the information from the risk scores, as we now explain.

Table D - DISMOD input data and data sources

| **Disease** | **First Parameter** | | **Second Parameter** | |
| --- | --- | --- | --- | --- |
|  | **Parameter** | **Source** | **Parameter** | **Source** |
| **Lung cancer** | Mortality | Mortality Statistics 2011[7] | Incidence | National Cancer Registry (2011)[8] |
| **Ischaemic Heart Disease** | Mortality | Mortality Statistics 2012[9] | Prevalence | Health Survey for England 2012[10] |
| **Stroke** | Mortality | Mortality Statistics 2012[9] | Prevalence | Health Survey for England 2012[10] |
| **Diabetes** | Standardised mortality rate | National audit of general practice 2011-12[11] | Prevalence | National audit of general practice 2011-12[11] |
| **Dementia** | Relative risk of mortality | Analysis of primary care data[12] | Incidence | CFAS II study[13] |

##

## Annual incidence of diseases

Annual incidence of CVD was computed using a combination of QRISK2 and the output from DISMOD. For an individual with age $a$, gender $g$ and remaining risk factors $x$ at a particular time, the simulation model requires the 1 year risk $q(a,g,x)$ of CVD. QRISK2, however, gives the 10-year risk, denoted $Q(a,g,x)$, while the DISMOD output gives the 1-year risk $d(a,g)$ for a person of age $a$ and gender $g$, but not broken down by other risk factors $x$. We therefore combined these two data sources as follows. The 10-year risk from DISMOD was calculated as

$$D\left( a,g \right)=\sum_{i=0}^{9} \left( 1-d\left( a+i-1, g \right) \right)^{i}d(a+i, g)$$

Therefore, conditionally on a person getting CVD within 10 years, the chance that this happens within the first year is

$$r\left( a,g \right)=\frac{d\left( a,g \right)}{D\left( a,g \right)}.$$

Hence, assuming this conditional probability is the same for all other risk factors $x$, the 1-year risk is estimated as

$$q\left( a,g,x \right)= r\left( a,g \right)Q\left( a,g,x \right).$$

Given that a CVD event is simulated, we then simulated whether the event is due to ischaemic heart disease (IHD) or stroke, using the relative incidence of each disease from DISMOD. In the following year, the QRISK2 score, 10 year risk and 1-year risk were recalculated in the same way.

The same method was used to calculate the annual probability of dementia onset. The CAIDE score (see Table E and Table F) estimates the 20-year risk of dementia, which we converted to a 1-year risk based on estimates of the average incidence in the population (by age and sex) estimated from routine data using DISMOD. For synthetic individuals aged 60 years or older, we assumed each synthetic individual stayed on the same risk percentile they were at when aged 60 years. A synthetic person’s increase in absolute risk with age is informed by the estimates derived from DISMOD using routine data. That is, risk continues to increase with age, but is not affected by changes to the CAIDE score after age 60 years.

**Table E: CAIDE risk score for predicting dementia in later life**

| Age |  |
| --- | --- |
| < 47 years  47-53years  > 53 years | 0  3  4 |
| Education |  |
| >9 years  7-9 years  0-6 years | 0  2  3 |
| Sex |  |
| Female  Male | 0  1 |
| Systolic BP |  |
| ≤140 mmHg  >140 mmHg | 0  2 |
| Body Mass Index |  |
| ≤30 kg/m^2^  >30 kg/m^2^ | 0  2 |
| Total Cholesterol |  |
| ≤6.5 mmol/L  >6.5 mmol/L | 0  2 |
| Physical Activity |  |
| Active  Inactive | 0  1 |

**Table F: CAIDE score and 20 year risk of dementia**

| **CAIDE score** | **20 year risk of dementia** |
| --- | --- |
| 0-5 | 1.0% |
| 6-7 | 1.9% |
| 8-9 | 2.4% |
| 10-11 | 7.4% |
| 12-15 | 16.4% |

Lung cancer incidence was based on estimates from DISMOD using routine data. Within DISMOD separate estimates for smokers and non-smokers were made, based on the proportion of incident cases of lung cancer attributable to smoking.[14] In this context, in the model, only current smokers are counted as smokers, while ex-smokers are considered as non-smokers.

Once an individual in the model has one of these diseases, we assumed that they had it for the rest of their life, and thus had a higher mortality and lower health-related quality of life.

Mortality

Mortality for a person with stroke, ischaemic heart disease, lung cancer, or dementia was modelled based upon estimated (using DISMOD) annual age, sex and disease-dependent Case Fatality Rates ${CFR}^{\left( r \right)}$ , defined as the expected number of deaths from each disease *r* divided by the number of person-years lived with that disease Assuming that these rates are constant within a year, the time to death from disease *r* for a person with disease *r* has an exponential distribution, thus the probability of death from disease *r* in one year for a person with disease *r* is $p^{(r)}= 1-exp(-{CFR}^{\left( r \right)})$*.*

Additionally we assumed that some people who had a CVD event died at the time of that event (i.e. the stroke or myocardial infarction was fatal). We assumed a probability of 0.5 that a CVD event was a myocardial infarction or a stroke. We assumed an instant fatality risk of 0.3 for both a myocardial infarction and a stroke.

The probability of death from CVD $p^{(CVD)}$ obtained from DISMOD, which includes the mortality from these events, is therefore adjusted so that it represents only non-event mortality for survivors of these events or people with CVD who have not experienced them yet. This adjusted mortality is ($p^{(CVD)}- p^{\left( I \right)})/(1- p^{\left( I \right)})$, where $p^{\left( I \right)}$is the probability of having a fatal event in a year, the top half of the fraction is the chance someone with CVD dies from something other than a fatal event within a year, and the bottom half is the chance of surviving a year without experiencing a fatal event.

Background mortality from other causes was estimated as follows. The probability of death from any cause was estimated as $\sum_{j} q_{j}p_{j}+ q_{\left\{ other \right\}}p_{\left\{ other \right\}}$, where $q_{j}$is the probability of death from modelled disease j, $p_{j}$is the prevalence of disease j, $q_{\left\{ other \right\}}$ is the probability of death from any other cause and $p_{\{other\}}$is the probability of having no modelled disease. Thus given (age-sex-dependent) data on all-cause mortality, cause-specific mortality and prevalence from DISMOD, we could deduce the other-cause mortality $q_{\left\{ other \right\}}$.

In the model, for every person at time *t* we simulated whether they died from other causes by time *t+1* using the probability $q_{\left\{ other \right\}}$. If they died, they were deemed to have died from another (non-modelled) cause. If they survived and had a disease, then we generated their disease-specific survival by *t+1* using the corresponding case fatality rate. If they had multiple diseases (less than 1% of people at each time point), then we simulated their disease-specific survival for each disease in turn in the order: stroke, IHD, dementia then lung cancer. The next disease in the list is only considered if they survive the previous disease.

We censor the model at 100 years of age, thus assuming that all individuals die at 100 years of age or earlier.

**2.3 Health Outcomes – calculation of QALYs**

QALYs were computed based upon EuroQol (EQ-5D) utility weights given in Table G making allowance for disease state, age, co-morbidities and income.[15]

Table G – EQ-5D codes used for diseases and socio-demographic covariates in the health check model to calculate QALYs

| Variable | **Disutility Weight (SE)** |
| --- | --- |
| Condition |  |
| LUNG CANCER (ICD9 162) | -0.1192(0.043) |
| IHD (ICD9 414) | -0.0627 (0.0131) |
| STROKE (ICD9 436) | -0.1171 (0.0121) |
| DEMENTIA (Clinical classification code 068) | -0.1917 (0.0141) |
| Multiple Conditions |  |
| Two | -0.0528 (0.0101) |
| Three | -0.0415 (0.0115) |
| Four | -0.0203 (0.0139) |
| Age (per year) | -0.003 (0.0002) |
| Least deprived four quintile groups (assume equate to low income, middle income and poor income) | 0.04 (0.006) |

Values taken from Sullivan et al, 2011 based on data from the 2000-2003 Medical Expenditure Panel Survey[15]

**3. Simulating Health checks**

**3.1 Eligibility**

Individuals in the model were eligible for a health check at each time point if they were aged 40-74, and had not yet been diagnosed with cardiovascular disease (CVD), diabetes or hypertension.

When individuals were diagnosed with hypertension or diabetes, or had a CVD event they became ineligible for health check in the future. Individuals could be diagnosed with diabetes or hypertension outside of the NHS Health Check Programme, as set out in Section 2.1.

**3.2 Attendance**

The NHS Health check programme was designed to operate on a rolling 5-year basis (i.e. each eligible individual was invited once every five years). We assumed that the probability of an eligible individual being offered a health check in any year was 0.197, based on published data.[16] Thus on average, a person will be offered health check once every five years.

For a person who is offered a health check, the probability they attend depends on their demographic characteristics. A baseline probability of attendance of 0.488 after invitation was assumed based on published data.[17] We assumed this was the probability of attendance for a person who is male, aged 50-59 years, white, living in the least deprived area, a non-smoker and who a QRISK2 score of five to 9.9. The probability of attendance for the other categories was calculated by converting this probability to odds, multiplying by odds ratios derived from the data in Table H and converted back to a probability.

We also assumed that 5% of ineligible individuals attended for a health check.

**3.3 Treatment assessment**

At the simulated health check visit, synthetic individuals were assumed to be offered one or more of four treatments:

1. A statin, with different offer probabilities dependent on whether QRISK2 is greater or less than 20%.
2. Hypertensive medication for anybody with a systolic blood pressure >140 mmHg at health check, with different offer probabilities depending on whether QRISK2 is greater or less than 20%.
3. Smoking cessation program for smokers
4. Weight management for those with a body mass index >30kg/m^2^ at assessment

The probabilities of treatment being offered are detailed in Table I.

**3.4 Treatment effect**

We simulated the effect of treatment by changing cardiovascular risk factors. Changes in risk factors as a result of treatment were based on data from published randomised controlled trials, summarised in Table I.

For people taking statins, total cholesterol levels was reduced, and HDL levels was increased, with a gender-dependent effect. Statins were also assumed to reduce the incidence of CVD through other

**Table H - Relative uptake of health checks among eligible population**

|  | **Numerator** | **Denominator** | **Percent**  **(95% CI)** | **Uptake ratio (95% CI)**  **relative to baseline category** |
| --- | --- | --- | --- | --- |
| Male | 832,227 | 102,555 | 12.3 (12.2, 12.4) | 1 |
| Female | 846,797 | 111,740 | 13.2 (13.1, 13.3) | 1.07(1.06, 1.08) |
| 40-49 | 806,199 | 72,903 | 9.04(8.98, 9.11) | 0.66(0.65, 0.67) |
| 50-59 | 499,725 | 68,428 | 13.7 (13.6, 13.8) | 1 |
| 60-69 | 373,100 | 72,964 | 19.6 (19.4, 19.7) | 1.41 (1.40, 1.42) |
| 70-74 |  |  |  | 1.52 (1.37, 1.64) |
| White | 1,065,171 | 185,082 | 17.4 (17.3, 17.5) | 1 |
| South Asian | 43,710 | 7,775 | 17.8 (17.4, 181) | 1.02 (1.00, 1.05) |
| Black | 32,807 | 5,659 | 17.3 (16.8, 17.7) | 0.99 (0.97, 1.02) |
| Other | 32,958 | 5,118 | 15.5 (15.1, 15.9) | 0.89(0.87, 0.92) |
| 1 - Least deprived | 336,174 | 41,423 | 12.3 (12.2, 12.4) | 1 |
| 2 | 334,996 | 40,342 | 12.0(11.9, 12.2) | 0.98 (0.97, 0.99) |
| 3 | 335,706 | 40,897 | 12.2 (12.1, 12.3) | 0.99 (0.98, 1.00) |
| 4 | 335,302 | 41,557 | 12.4 (12.3, 12.5) | 1.01 (0.99, 1.02) |
| 5 - Most deprived | 334,652 | 49,974 | 14.9 (14.8, 15.1) | 1.21 (1.20, 1.23) |
| Non-smoker | 1250885 | 176212 | 14.1 (14.0, 14.2) | 1 |
| Smoker | 347842 | 37808 | 10.9 (10.8, 11.0) | 0.77 (0.76, 0.78) |
| QRISK2<5 | 243047 | 47794 | 19.7 (19.5, 19.8) | 0.71 (0.70, 0.72) |
| QRISK2 5-9.9 | 158393 | 43687 | 27.6 (27.4, 27.8) | 1 |
| QRISK2 10-14.9 | 87758 | 32452 | 37.0 (36.7, 37.3) | 1.34 (1.32, 1.36) |
| QRISK2 15-19.9 | 53995 | 22639 | 41.9 (41.4, 42.4) | 1.52 (1.49, 1.55) |
| QRISK2 20+ | 52771 | 24869 | 47.1 (46.7, 47.6) | 1.71 (1.68, 1.74) |

Based on Robson et al, *2016*.[17] The estimates for 60-74 years of age, were presented as a single estimate by Robson et al. (with a combined value of 1.43). We have split this estimate into two age groups, drawing on the relative size of those groups in Chang et al.[18]

(non-cholesterol mediated) mechanisms, and consequently we made and a direct adjustment to the QRISK2 score to reflect this, estimated by calibrating the modelled reduction in CVD event rates over 5 years achieved by statin treatment to equal the value observed in trial data (see Table I).

Treatments may be given outside of the health check process in either trajectory, but these are assumed to be the same in both the background and the “treated” trajectories, so that the difference between the trajectories captures the effect of treatment specifically delivered via health check.

For people taking anti-hypertensive medication, we assumed that both systolic and diastolic blood pressure were lowered, with effects that depended on age and gender.

A proportion of those referred to smoking cessation were assumed to have quit after one year.

Everyone attending at least one session of weight management lost weight by a year, resulting in lowered BMI. We assumed weight lost was gradually regained over the next five years.

For all treated individuals, we assumed a background (counterfactual) trajectory for each risk factor, without treatment via a health check. This simulated based on the ELSA data, as described in Section 2.1. For individuals treated with statins, anti-hypertensive medication or weight management via a health check, a treated trajectory is computed by shifting the background trajectory during the period on treatment. For example, at each time point when a woman is on statins, her cholesterol level will be exactly 1.16 mmol/L less than her background cholesterol level at the equivalent time point.

Under this approach some people's cholesterol will increase after receiving statins, the increase will be less than it would have been had they had not started a statin (i.e. be less than their background trajectory). An alternative approach would have been to reduce cholesterol by the one-year effect observed in the trials, but there was a lack of appropriate data, particularly over the longer-term, to model cholesterol reductions in this way. Another alternative would have been to estimate future trajectories by sampling from ELSA. However lower cholesterol values observed in ELSA will tend to improve through time, due to regression to the mean, so such an approach would likely under estimate the benefits of statin treatment.

For individuals who had quit smoking after attending a smoking cessation program, their risk of relapse and future smoking trajectory was computed by matching to individuals in the ELSA data, as set out in Section 2.1.

We also modelled adherence to treatment. These assumptions were included in Table I. We also assumed that 5% of individuals taking statins or anti-hypertensive medication were assumed to stop taking them each year. Individuals who were not adherent to treatment returned to their background trajectory, i.e. we assumed no benefit from past treatment on risk factors after cessation of treatment.

Table I – Summary of key assumption within the model

| **Part of model** | **Parameter** | **Data source / Assumption** |
| --- | --- | --- |
| **A. Baseline population** | **A.1.** Socio-demographic composition and prevalence of cardiovascular risk factors at baseline | Health Survey for England 2009-2012  Missing data assumed missing at random |
|  | **A.2.** Prevalence of cardiovascular disease at baseline | Estimated based on routine data using DISMOD (see Table D). Diseases given to the synthetic individuals with the highest risk scores. |
| **B. Health over time** | **B.1.** Trajectories of health characteristics over time | English Longitudinal Study of Ageing (ELSA) 1998-2012.  Synthetic individual matched to a similar ELSA individual, and characteristic 2 or 4 years later drawn from the next ELSA observation for that individual, with linear interpolation  Assume people are ex-smokers if there are two consecutive records of not smoking.  Smoking quit rate assumed to be 5% instead of the 6.5% in ELSA, and relapse rate assumed to be 37% over 10 years.[2] |
|  | **B.2.** Incidence of cardiovascular disease and dementia | 10-year risk of CVD calculated using QRISK2 score and dementia using CAIDE score, based on current simulated values of risk factors.  10-year and 20-year risk converted to annual risk based on routine data.  Relative incidence of IHD and stroke based on routine data. |
|  | **B.3.** Incidence of lung cancer in smokers and non-smokers | Based on cancer registry data for the number of cases of lung cancer, and published estimates of the proportion of lung cancer attributable to smoking in the UK by age and sex, which ranges from 25% to 89%,.[14], we estimated the number of cases of lung cancer in smokers and non-smokers. Using population estimates we then estimated incidence. Annualised estimates of incidence were made using DISMOD. |
|  | **B.4.** Case fatality from each disease, and mortality from other causes | Estimated from routine data using DISMOD.  Instant fatality rates from myocardial infarction 32% for men and 30% for women[19]Instant fatality rates from stroke: 24%, 17% for men and women respectively aged 80 and over, and 7%, 5% for men and women respectively aged under 80[20]  Probability that an IHD event is a myocardial infarction 58% for men, and 44% for women[21]  Probability that a “stroke” event is a full stroke (as opposed to transient ischaemic attack) 60%,[22] |
| **C. Who gets a Health check** | **C.1.** Proportion of eligible population offered health check | 19.7% per year.[16] |
|  | **C.2.** Proportion of people offered health check who attend | 48%[16] assumed to apply to a white man aged 50-59 years who lives in the least deprived area is a non-smoker and has a QRISK2 score of 5 to 9.9. |
|  | **C.2.** Relative uptake of health check by different characteristics, among eligible people | Relative uptake by age, sex, ethnicity, deprivation, smoking status and QRISK2 score.[17] Further breakdown of uptake by age based on Chang et al.[18] Further detail given in Table H. |
|  | **C.3.** Proportion of people getting health check who are not eligible on the basis of a chronic condition | 5% (95% credible interval 2% to 8%): estimated by study team: no data available. |
|  | **C.4.** Relative uptake of health check by ineligible people | GP attendance rate by age & sex,[23] also assuming GP attendance rates are higher for South Asians. (HSE 2005, our analyses), and that the predictors of GP attendance in general are the same as those of having a health check. |
| **D. Who gets treated** | **D.1.** Proportion of smokers at health check who are referred to smoking cessation therapy | 3.6% (95% CI 3.3%, 3.9%).  Deduced knowing 6.8% of smokers (2571/37808) who had a health check were referred to smoking cessation, compared to 3.2% (9944/310,034) of smokers who do not have a health check.[17] |
|  | **D.2.** Proportion of obese people (BMI ≥ 30) at health check who are referred to weight management interventions | 27.5 % (95% CI 26.9%, 28.1 %).  Deduced knowing 38.7% (12430/32133) of obese people and who had a health check referred to weight management, compared to 11.2% (4441/39774) of obese people who did not have health check.[17]  Only considers weight management, not the additional 31.1% of obese people who were referred to exercise, a group which is assumed to overlap substantially. |
|  | **D.3.** Proportion of (a) high-risk and (b) low-risk individuals (using QRISK2) who receive statins | Robson et al. 2014,[24] Robson et al. 2016.[17]  QRISK2<20%: 2.05% (95%CI 1.97, 2.13) additional statins prescriptions in health check attendees versus non-attenders  QRISK2>=20%: 14.23% (95%CI 13.71, 14.76) additional statins prescriptions in health check attendees versus non-attenders  ‘Newly receive statins’ defined as getting two or more prescriptions |
|  | **D.4.** Proportion of people with high blood pressure who receive anti-hypertensives | Robson et al. 2014,[24] Robson et al. 2016.[17]  QRISK2<20%: 1.54% (1.46, 1.62) additional anti-hypertensives prescriptions in health check attendees versus non-attenders  QRISK2>=20%:2.48% (95%CI 2.05, 2.90) additional anti-hypertensives prescriptions in health check attendees versus non-attenders  Only individuals with hypertension at health check (defined as systolic blood pressure greater than140mmHg) assumed to get anti-hypertensive treatment in either case.  ‘Newly receive anti-hypertensives’ defined as getting two or more prescriptions |
| **E. Adherence rates** | **E.1.** Smoking cessation adherence | Assume that 100% of patients referred ‘adhere’ to treatment as the treatment effectiveness estimates include those who are non-adherent. |
|  | **E.2.** Weight management programme adherence | 50% assumed to attend at least one session (personal communication from Amy Ahern based on WRAP trial). 95% credible interval of 30% to 70% from our assumption. |
|  | **E.3.** Statins adherence | 50% adherence to initial prescription (with 95% credible interval of 40% to 60%, from our assumption).[25–27]  An additional 5% (95% CI 3% to 7%) of people taking statins are assumed to stop taking them each year (our assumption). |
|  | **E.4.** Antihypertension adherence | 55% adherence (with 95% credible interval of 45% to 65%, from our assumption).[25,27,28]  An additional 5% (95% CI 3% to 7%) of people on AHTs are assumed to stop taking them each year (our assumption). |
| **F. Treatment effectiveness** | **F.1.** Smoking cessation | 14.6% (95%CI 13.1, 16.1) of those who are referred have quit at 1 year,[29] consistent with Agboola et al.[30] |
|  | **F.2.** Weight management effectiveness | BMI change of -1.5kg/m^2^ by 1 year for everyone attending at least one session.[31]  Lost weight assumed to be regained over 5 years, with BMI changes of -1.5, -0.9, -0.6, -0.3 and 0 at 1, 2, 3 4 and 5 years after health check, respectively. |
|  | **F.3.** Statins effectiveness | Mean change of -1.22 (95%CI -1.19, -1.26) men, -1.16 (95%CI -1.10, -1.23) women in total cholesterol at 1 year.[32]  Mean increase in HDL at 1 year 0.04 (0.028, 0.052) for men, and 0.036 (0.012, 0.060) for women, personal communication from study authors.[32]  Relative rate of cardiovascular events during 5 year follow-up 0.84 (0.78 to 0.91) and 0.78 (0.75, 0.81).[32] |
|  | **F.4.** Anti-hypertensive medication effectiveness | Assume that those under 55 years (both sexes), use an ACE inhibitor. Heran et al. 2008 indicates an average change for Ramipril of -6.29/-4.14 mmHg (95%CI: -9.26 to -3.32/-5.81 to -2.48) DBP.[33]  Assume those aged 55 years and over use calcium channel blockers. Turnbull et al. 2008 reports an average change (relative to placebo) of -7.6/-3.1 mmHg (95% CI -7.95 to -7.25/-2.75 to -3.45) for men, and -9.0/-3.5 mmHg (95%CI -8.68 to -9.32/-3.18 to -3.82) for women.[34] |
|  | **All** | Treatment effects assumed to be the same for everybody receiving the treatment, unless otherwise stipulated. |
| G. Trends in CVD (Sensitivity analysis) | **G.1.** IHD incidence | The acute myocardial infarction rate declined at an average annual rate of 4.8% (95% CI: 3.0% to 6.5%) for men and 4.5% (1.7% to 7.1%) for women between 2002 and 2010. We applied these rates to all IHD.[19] |
|  | **G.2.** Stroke incidence | 30% decline over nine years, from 1999-2008 based on analysis of GP data, equivalent to 4% decline per year.[35] |
|  | **G.3 IHD case fatality** | The acute myocardial infarction case fatality rate declined at an average annual rate of 3.6% (3.4% to 3.7%) for men and 4.2% (4.0% to 4.3%) for women between 2002 and 2010. We applied these rates to all IHD.[19] |
|  | **G.4. Stroke case fatality** | 56-day mortality for first stroke for men and women reduced from 21% to 12% over nine years from 1999-2008 based on analysis of GP data, equivalent to 6% decline per year.[35] |
|  | **G.5. Time period for trends** | Given the data on which the trends is based are relatively short-term (8-9 years) and concern that incidence of CVD may rise due to the rising prevalence of obesity and diabetes, we have modelled the trends continuing for another 20 years, before plateauing. We also note the persistence of the trends is likely to be due in part to better uptake and use of existing treatments, and we do not know the extent to which new treatments or management strategies can impact these future trends. |

**Table J: Key assumptions that may result in over- or under- of overall health benefit**

| **May result in underestimate**   - Not modelled impact of smoking cessation on COPD and other cancers - Not included other (non-cardiovascular) benefits of weight loss - Not modelled impact of brief interventions to reduce alcohol consumption - Assumed obesity does not increase - Assumed a high rate of medication discontinuation (50% initially and 5% per annum thereafter) |
| --- |
| **May result in overestimate**   - Not modelled decline in CVD incidence - Not modelled decline in smoking prevalence - Not made allowance for new treatments - Inclusion of dementia if reducing cardiovascular risk does not reduce dementia risk - Not modelled side effects of treatments |

**4. Uncertainty quantification**

A synthetic population of size N was simulated. The expected survival (and similar quantities) under the model was calculated as the empirical mean survival for the population of size N, but the estimate of this mean will be subject to a small error due to the finite size of the synthetic population, which we will call “computational” uncertainty or error.

A further source of uncertainty arises from incomplete knowledge of the parameters. This can be described as “statistical uncertainty” and was accounted for by undertaking a probabilistic sensitivity analysis. In this approach, model input parameters were assigned probability distributions instead of fixed values. The simulation of the population of size N was repeated M times, each time with a different set of parameter values, with simulated values being drawn from the appropriate distributions. Additionally, during each of the M simulations, a different random number seed (arbitrarily fixed to r at simulation number r out of M to ensure reproducibility) is used, which ensures that by sampling the baseline population from HSE as in Section 2, and by sampling from the ELSA data to simulate risk factor trajectories as in Section 2.1, uncertainty due to the finite size of HSE and ELSA is accounted for. This is a form of bootstrap estimation.

We took N=200000 and M=100, and ran the simulations in parallel on 80 cores of a high-performance computing cluster, which took around 14 hours. N and M were chosen to minimise computational error within the available computing time. The uncertainty or credible intervals presented in the manuscript for expected health gains under each scenario represent the 2.5th and 97.5th percentiles of the M estimates of the empirical mean produced when undertaking M simulations. Thus the intervals nominally represent statistical uncertainty, but are also affected by a small computational error. Using methods described by O’Hagan et al.[36], we estimated that the computational standard errors were typically around 10% of the statistical error.

The probability distributions used for probabilistic sensitivity analysis were derived from published data where possible. For parameters representing probabilities, beta distributions were used, and for theoretically-unbounded quantities such as changes in risk factors after treatment, normal distributions were used, with parameters derived from the confidence or credible intervals stated in Table I. For the DISMOD output, uncertainty was not considered.

**4.1 Value of Information analysis**

We used the results of the uncertainty analysis to estimate the potential value of gaining further information on the uncertain parameters. Specifically, we estimated the expected reduction in uncertainty in each of two key model outputs $Y$:

- days of quality-adjusted life gained from health checks compared to a scenario with no health checks, estimated as 3.8 days (95% interval 3.0 - 4.7)
- days of quality-adjusted life gained from feasible increases to NHS Health Check eligibility, uptake and treatment, compared to current NHS Health Check practice, estimated as 10 days (95% interval 8.2 - 12.2 days)

if we were to learn one of the uncertain parameters $\theta$precisely. We repeated this for each parameter $\theta$with uncertainty intervals listed in Table I. Specifically, we estimated $S_{\theta}=\sqrt{E_{\theta}(\mathrm{var}(Y|\theta))}$, the predicted standard deviation of the uncertainty distribution of $Y$after learning $\theta$. This is related to the “expected value of partial perfect information” and computed using methods described by Jackson et al [https://arxiv.org/abs/1703.08994] based on regression of the sampled model outputs on the sampled model inputs.

**5. References**

1 Hyland A, Li Q, Bauer JE, *et al.* Predictors of cessation in a cohort of current and former smokers followed over 13 years. *Nicotine Tob Res* 2004;**6 Suppl 3**:S363-9.http://www.ncbi.nlm.nih.gov/pubmed/15799599 (accessed 20 Feb2017).

2 Centers for Disease Control and Prevention (CDC). Quitting smoking among adults--United States, 2001-2010. *MMWR Morb Mortal Wkly Rep* 2011;**60**:1513–9.http://www.ncbi.nlm.nih.gov/pubmed/22071589 (accessed 20 Feb2017).

3 Hawkins J, Hollingworth W, Campbell R. Long-Term Smoking Relapse: A Study Using the British Household Panel Survey. *Nicotine Tob Res* 2010;**12**:1228–35. doi:10.1093/ntr/ntq175

4 Hippisley-Cox J, Coupland C, Vinogradova Y, *et al.* Predicting cardiovascular risk in England and Wales: prospective derivation and validation of QRISK2. *BMJ* 2008;**336**:1475–82. doi:10.1136/bmj.39609.449676.25

5 Collins GS, Altman DG. An independent and external validation of QRISK2 cardiovascular disease risk score: a prospective open cohort study. *BMJ* 2010;**340**.

6 Kivipelto M, Ngandu T, Laatikainen T, *et al.* Risk score for the prediction of dementia risk in 20 years among middle aged people: a longitudinal, population-based study. *Lancet Neurol* 2006;**5**:735–41. doi:10.1016/S1474-4422(06)70537-3

7 Office for National Statistics. Deaths registered in England and Wales, 2011. Newport, Wales: 2013. http://www.ons.gov.uk/ons/publications/re-reference-tables.html?edition=tcm%3A77-27772730

8 Office for National Statistics. Cancer Registrations in England, 2011. Newport, Wales: 2013. http://www.ons.gov.uk/ons/publications/re-reference-tables.html?edition=tcm%3A77-302299

9 Office for National Statistics. Mortality Statistics: Deaths registered in England and Wales, 2012. London: 2014.

10 Scholes S, Mindell J. Health Survey for England 2012 - Physical activity in adults. Leeds: 2013. http://www.hscic.gov.uk/catalogue/PUB13218/HSE2012-Ch2-Phys-act-adults.pdf

11 Health & Social Care Information Centre. National Diabetes Audit 2011-12 Report 2: Complications & Mortality. Leeds: 2013. http://digital.nhs.uk/catalogue/PUB12738/nati-diab-audi-11-12-mort-comp-rep.pdf

12 Rait G, Walters K, Bottomley C, *et al.* Survival of people with clinical diagnosis of dementia in primary care: cohort study. *BMJ* 2010;**341**:c3584.http://www.pubmedcentral.nih.gov/articlerender.fcgi?artid=2917003&tool=pmcentrez&rendertype=abstract (accessed 12 Jun2014).

13 Matthews FE, Arthur A, Barnes LE, *et al.* A two-decade comparison of prevalence of dementia in individuals aged 65 years and older from three geographical areas of England: results of the Cognitive Function and Ageing Study I and II - PIIS0140673613615706.pdf. Lancet. 2013;**382**:1405–12.http://download.thelancet.com/pdfs/journals/lancet/PIIS0140673613615706.pdf?id=410a13c7e856fa01:-537181ea:142b8fe4bec:1b371386088006979 (accessed 5 Dec2013).

14 Parkin DM. 2. Tobacco-attributable cancer burden in the UK in 2010. *Br J Cancer* 2011;**105 Suppl**:S6–13. doi:10.1038/bjc.2011.475

15 Sullivan PW, Slejko JF, Sculpher MJ, *et al.* Catalogue of EQ-5D Scores for the United Kingdom. *Med Decis Mak* 2011;**31**:800–4. doi:10.1177/0272989X11401031

16 NHS England. NHS Health Checks: National Data. http://www.healthcheck.nhs.uk/interactive map/ (accessed 16 Mar2015).

17 Robson J, Dostal I, Sheikh A, *et al.* The NHS Health Check in England: an evaluation of the first 4 years. *BMJ Open* 2016;**6**:e008840. doi:10.1136/bmjopen-2015-008840

18 Chang KC-M, Soljak M, Lee JT, *et al.* Coverage of a national cardiovascular risk assessment and management programme (NHS Health Check): Retrospective database study. *Prev Med (Baltim)* 2015;**78**:1–8. doi:10.1016/j.ypmed.2015.05.022

19 Smolina K, Wright FL, Rayner M, *et al.* Determinants of the decline in mortality from acute myocardial infarction in England between 2002 and 2010: linked national database study. *BMJ* 2012;**344**:d8059. doi:10.1136/bmj.d8059

20 Lee S, Shafe ACE, Cowie MR. UK stroke incidence, mortality and cardiovascular risk management 1999-2008: time-trend analysis from the General Practice Research Database. *BMJ Open* 2011;**1**:e000269. doi:10.1136/bmjopen-2011-000269

21 British Heart Foundation Centre On Population Approaches For Non-Communicable Disease Prevention. Cardiovascular Disease Statistics 2015. London: 2016.

22 Luengo-Fernandez R, Gray AM, Rothwell PM, *et al.* A population-based study of hospital care costs during 5 years after transient ischemic attack and stroke. *Stroke* 2012;**43**:3343–51. doi:10.1161/STROKEAHA.112.667204

23 Hippsley-Cox J, Vinogradova Y. Trends in consultation rates in general practice 1995 to 2008. Leeds: 2009. https://catalogue.ic.nhs.uk/publications/primary-care/general-practice/tren-cons-rate-gene-prac-95-09/tren-cons-rate-gene-prac-95-09-95-08-rep.pdf

24 J R, Dostal I, Sheikh A, *et al.* NHS Health Check National Evaluation 2009-2013. 2014.

25 Naderi SH, Bestwick JP, Wald DS. Adherence to drugs that prevent cardiovascular disease: meta-analysis on 376,162 patients. *Am J Med* 2012;**125**:882–7.e1. doi:10.1016/j.amjmed.2011.12.013

26 Lemstra M, Alsabbagh MW. Proportion and risk indicators of nonadherence to antihypertensive therapy: a meta-analysis. *Patient Prefer Adherence* 2014;**8**:211–8. doi:10.2147/PPA.S55382

27 Faculty of Public Health. Response from the Faculty of Public Health: Technical Consultation on Economic Modelling of a Policy of Vascular Checks. London: 2008. http://www.fph.org.uk/uploads/DH_vascular_checks_0808.pdf

28 Chowdhury R, Khan H, Heydon E, *et al.* Adherence to cardiovascular therapy: a meta-analysis of prevalence and clinical consequences. *Eur Heart J* 2013;**34**:2940–8. doi:10.1093/eurheartj/eht295

29 Ferguson J, Bauld L, Chesterman J, *et al.* The English smoking treatment services: one-year outcomes. *Addiction* 2005;**100**:59–69. doi:10.1111/j.1360-0443.2005.01028.x

30 Agboola SA, Coleman TJ, McNeill AD. Relapse prevention in UK Stop Smoking Services: a qualitative study of health professionals’ views and beliefs. *BMC Health Serv Res* 2009;**9**:67. doi:10.1186/1472-6963-9-67

31 Stubbs RJ, Pallister C, Whybrow S, *et al.* Weight Outcomes Audit for 34,271 Adults Referred to a Primary Care/Commercial Weight Management Partnership Scheme. *Obes Facts* 2011;**4**:1–1. doi:10.1159/000327249

32 Cholesterol Treatment Trialists’ (CTT) Collaboration, Fulcher J, O’Connell R, *et al.* Efficacy and safety of LDL-lowering therapy among men and women: meta-analysis of individual data from 174 000 participants in 27 randomised trials. *Lancet* 2015;**385**:1397–405. doi:10.1016/S0140-6736(14)61368-4

33 Heran BS, Wong MM, Heran IK, *et al.* Blood pressure lowering efficacy of angiotensin converting enzyme (ACE) inhibitors for primary hypertension. In: Heran BS, ed. *Cochrane Database of Systematic Reviews*. Chichester, UK: : John Wiley & Sons, Ltd 2008. CD003823. doi:10.1002/14651858.CD003823.pub2

34 Turnbull F, Woodward M, Neal B, *et al.* Do men and women respond differently to blood pressure-lowering treatment? Results of prospectively designed overviews of randomized trials. *Eur Heart J* 2008;**29**:2669–80. doi:10.1093/eurheartj/ehn427

35 Lee S, Shafe ACE, Cowie MR. UK stroke incidence, mortality and cardiovascular risk management 1999-2008: time-trend analysis from the General Practice Research Database. *BMJ Open* 2011;**1**:e000269. doi:10.1136/bmjopen-2011-000269

36 O’Hagan A, Stevenson M, Madan J. Monte Carlo probabilistic sensitivity analysis for patient level simulation models: efficient estimation of mean and variance using ANOVA. *Health Econ* 2007;**16**:1009–23. doi:10.1002/hec.1199
